# Effective Design Strategy of Small Bipolar Molecules through Fused Conjugation toward 2.5 V Based Redox Flow Batteries

**DOI:** 10.1021/acsenergylett.2c00198

**Published:** 2022-03-08

**Authors:** Yue Liu, Gaole Dai, Yuanyuan Chen, Ru Wang, Huamei Li, Xueliang Shi, Xiaohong Zhang, Yang Xu, Yu Zhao

**Affiliations:** † Institute of Functional Nano & Soft Materials (FUNSOM), Jiangsu Key Laboratory for Carbon-based Functional Materials & Devices, Soochow University, 199 Renai Road, Suzhou, Jiangsu 215123, People’s Republic of China; ‡ Department of Chemistry, University College London, 20 Gordon Street, London WC1H 0AJ, U.K.; § College of Materials, Chemistry and Chemical Engineering, Hangzhou Normal University, 2318 Yuhangtang Road, Hangzhou, Zhejiang 311121, People’s Republic of China; ∥ Shanghai Key Laboratory of Green Chemistry and Chemical Processes, School of Chemistry and Molecular Engineering, East China Normal University, 500 Dongchuan Road, Shanghai 200062, People’s Republic of China

## Abstract

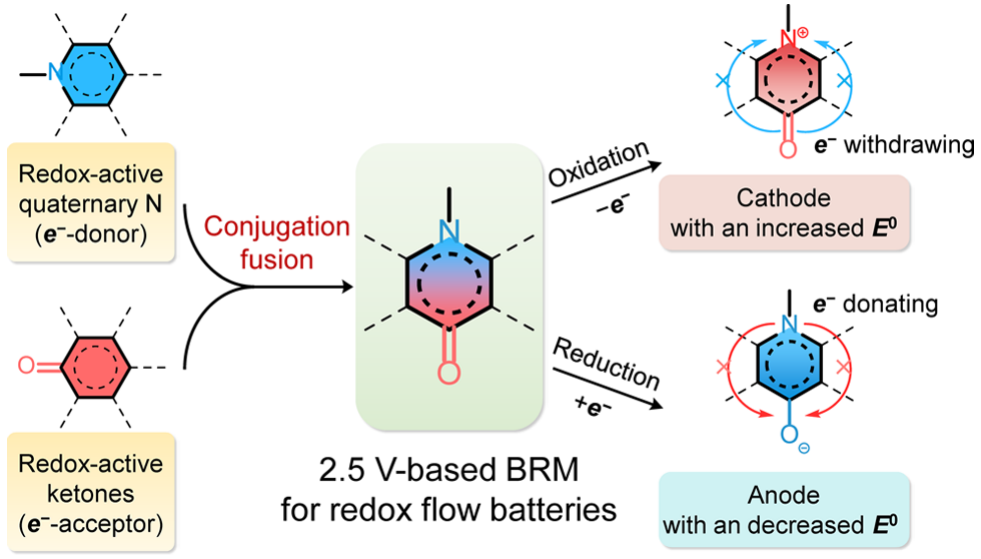

Using bipolar redox-active molecules (BRMs) as active materials is a practical way to address electrolyte crossover and resultant unpredictable side reactions in redox-flow batteries. However, the development of BRMs is greatly hindered by difficulties in finding new molecules from limited redox-active moieties and in achieving high cell voltage to compete with existing flow battery chemistries. This study proposes a strategy for design of high-voltage BRMs using fused conjugation that regulates the redox potential of integrated redox-active moieties. As a demonstration, quaternary N and ketone redox moieties are used to construct a new BRM that shows a prominent voltage gap with good electrochemical stability. A symmetrical redox-flow cell based on this molecule exhibits a high voltage of 2.5 V and decent cycling stability. This study provides a general strategy for designing new BRMs that may enrich the cell chemistries of organic redox-flow batteries.

Redox-flow batteries (RFBs) are considered to be a promising storage technology for grid-scale electrical energy storage due to the flexibility of configuring cells and the ability to decouple power and energy density in comparison to other battery technologies using solid electrodes.^1−3^ Although RFBs have achieved a remarkable improvement in the past few decades, obstacles such as relatively low energy density and electrolyte crossover need to be addressed before they can proceed to practical implementation.^4−6^ On the one hand, nonaqueous solvents are preferable over the aqueous counterparts to improve energy density, because the former has a broader electrochemical potential window than the latter, which is essential for the cell to reach a high output voltage.^7−9^ Moreover, the great number of nonaqueous-soluble redox-active organic molecules (ROMs) significantly broaden the range to choose active materials. The benefit of using ROMs as active materials arises from their elemental sustainability, structural diversity, and more importantly, structural tunability that is the key to enhance the (electro)chemical stability and tailor the redox potential of RFBs via molecular engineering strategies.^10−13^ On the other hand, crossover can cause electrolyte contamination, which inevitably lowers the utilization ratio of active materials and may trigger unpredictable side reactions between electrolyte constituents.^14^

A practical way to address the above challenges is to use bipolar redox-active molecules (BRMs). They are different from traditional ROMs, because they can be either oxidized to a positively charged state or reduced to a negatively charged state, with the features of both reduction and oxidation reactions being independent and reversible.^15^ As a result, the two half-compartments of an RFB can utilize the same redox-active molecule and electrolyte, i.e., a symmetrical RFB.^16,17^ This configuration can mitigate the challenge of crossover by eliminating the chemical concentration gradient in the discharge state.^18^ Even if the charging species permeate across the separator during cell operation, a symmetrical RFB can return to its initial state to avoid permanent degradation caused by an internal shuttle effect, which can theoretically extend the cell lifetime and enhance the utilization efficiency of BRMs.^17,19^ Due to these remarkable characteristics, symmetrical RFBs hold great prospects for grid-scale electrical energy storage. So far, several strategies have been developed to construct symmetrical RFBs, including forming bipolar eutectic mixtures (Figure S1a),^20−23^ combining different redox-active moieties through covalent bonding (Figure S1b),^24−27^ and physically mixing anode- and cathode-active molecules to form an electrolyte (Figure S1c).^28,29^ Although these strategies are highly promising in addressing the challenge of electrolyte contamination, they all have obvious weaknesses. Bipolar eutectic mixtures generally exhibit a high viscosity that significantly reduces the diffusion rate of active molecules, so that the resulting electrolytes could only be operated at a relatively low current density.^30,31^ Both physically mixed redox-active moieties and covalently bonded anode- and cathode-active molecules would not change the electrochemical characteristics of the original redox-active moieties, while complicated fabrication procedures raise the manufacturing cost.^32−34^ Furthermore, current studies on BRMs are limited by the fact that BRMs are composed of previously known redox-active molecules or their combinations, and BRMs that are capable of delivering an output voltage of greater than 2.5 V have yet to be demonstrated.^27,35−39^ Therefore, it is highly desirable to develop fundamentally new BRM structures with a high output voltage and regulatable electrochemical characteristics with the utmost compatibility with nonaqueous RFBs.

The electrochemical characteristics of ROMs are directly determined by the electron density distribution around the redox-active moiety and can be regulated by incorporating electron-withdrawing and -donating groups into the molecules.^40^ As is known, p-type molecules are regarded as electron donors because they prefer to lose electrons in redox reactions, whereas n-type molecules are regarded as electron acceptors because they prefer gaining electrons in redox reactions.^41^ When a p-type moiety and a n-type moiety are sufficiently close to each other, at which point they are fused to a great extent through conjugation sharing,^42^ it is possible to simultaneously and synergically regulate the redox potentials of the moieties due to the direct electronic perturbation between them and form a new type of BRM. In this study, we demonstrate an effective and general design strategy of BRMs by fusing p- and n-type redox moieties in the same conjugated structure through synergized electron delocalization and inductive effects. As a demonstrator, we prepared a BRM, 11-methoxy-9*H*-quinolino[3,2,1-*kl*]phenothiazin-9-one, denoted QPT-OMe, and applied it as both anode and cathode materials for a symmetrical RFB. The battery delivers an impressive discharge voltage of ∼2.5 V, which is superior to most of the reported symmetrical RFBs based on BRMs. Moreover, a density functional theory (DFT) study reveals that, in comparison with the pristine p*-* and n*-*type moieties, QPT-OMe shows an extended conjugation that enables sufficient charge delocalization and stable intermediates in the redox reactions.^43^ In addition to the enhanced discharge voltage, our design strategy of BRMs eliminates the weaknesses of previous strategies and the issue of electrolyte crossover in conventional RFBs. We believe the strategy might open a new avenue to design BRMs and in a wide sense benefit the performance improvement of RFBs.

Figure 1a illustrates our design strategy of BRMs by using a quaternary N (electron donor) and a ketone (electron acceptor) as the redox-active moieties because they have been widely adopted in organic batteries and RFBs. Upon conjugation fusion, the molecular orbital would be reorganized in comparison to each individual redox-active moiety. Once the two redox-active moieties are fused into the same conjugation, the electron density around the N atom is reduced due to the electron-withdrawing effect from the *para* ketone moiety, while the electron density around the ketone moiety is increased due to the electron-donating effect from the *para* quaternary N that possesses a lone pair electron in the *p* orbital perpendicular to the conjugated plane.^44,45^ When an oxidation reaction occurs, a higher potential is needed to remove one electron from the *p* orbital of N because the negative charge compensation is obstructed by the presence of the electron-withdrawing ketone moiety, resulting in an increase in oxidation voltage. Similarly, when a reduction reaction occurs, a lower potential is needed for the ketone moiety to gain one electron because negative charge compensation is contributed by the presence of the electron-donating quaternary N moiety, resulting in a decrease in reduction voltage. As a result, when such a kind of BRM is used as both the cathode and anode material in a symmetrical RFB, the potential separation of the two half-reactions is enlarged; hence, the output voltage of the RFB is increased and the intermediates of the BRM could be stabilized by the intensified electron delocalization.

**Figure 1:**
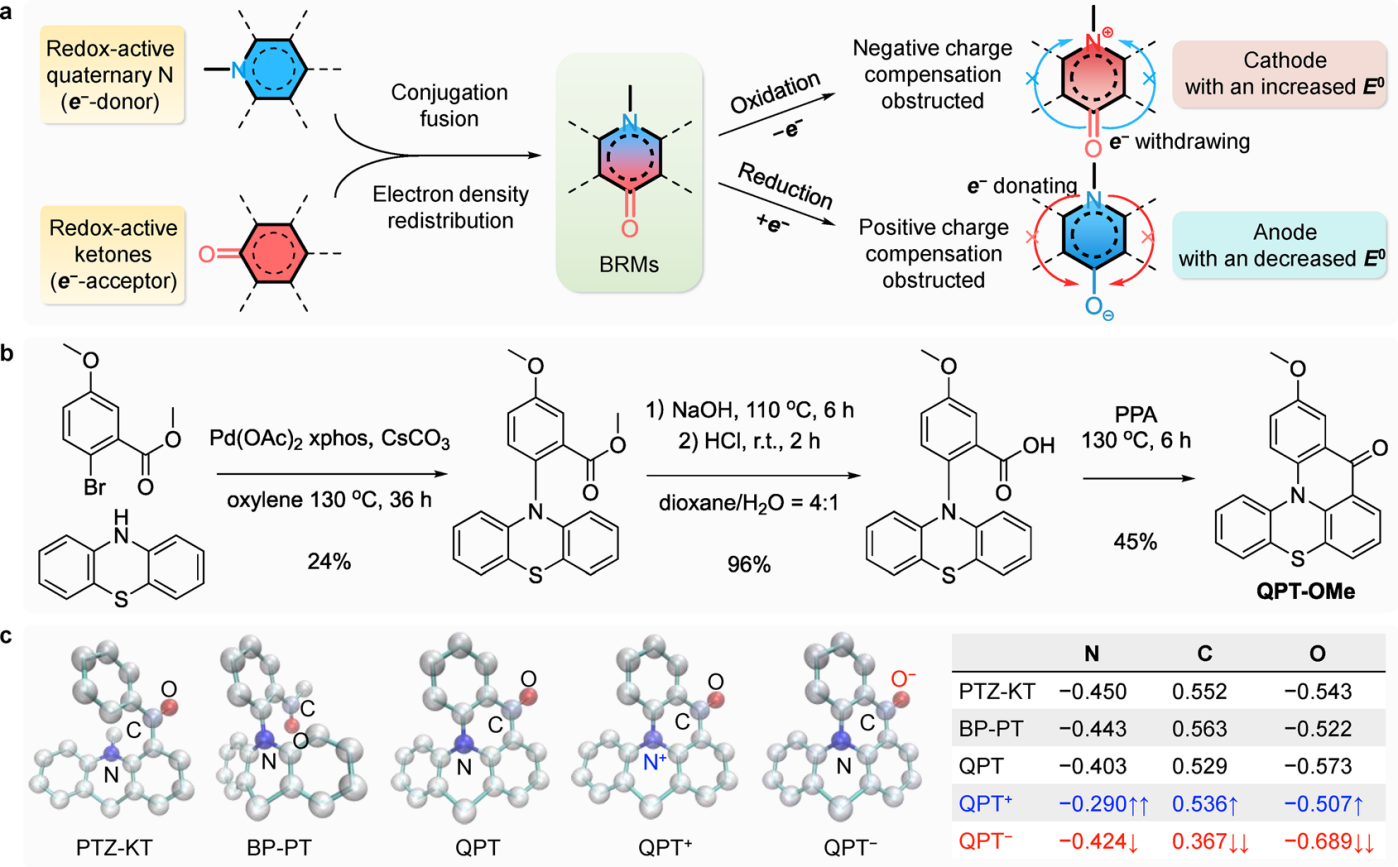
Design strategy, synthesis, and electron density redistribution in selected BRMs with fused conjugation. (a) Molecular design strategy of BRM using quaternary N and a ketone as the redox-active moieties. (b) Synthetic routes for QPT-OMe. (c) NPA study of electron density redistribution in PTZ-KT, BP-PT, QPT, and its redox intermediates. The NPA charges of other atoms are not shown for clarity.

On the basis of this strategy, we designed the QPT-OMe molecule by fusing a ketone as the electron-withdrawing moiety and a quaternary N as the electron-donating moiety into a conjugated system. As is shown in Figure 1b, QPT-OMe was synthesized using inexpensive and commercially available methyl 2-bromo-5-methoxybenzoate and 10*H*-phenothiazine through Pd-catalyzed coupling, followed by an acidification reaction after hydrolysis and intramolecular acylation effecting cyclization (see synthetic procedures in the Supporting Information). In addition, in order to achieve a high solubility in a nonaqueous electrolyte,^46^ an ether chain was grafted to the QPT core (see synthetic procedures in the Supporting Information) and the obtained BRM, 11-(2-(2-(2-methoxyethoxy)ethoxy)ethoxy)-9*H*-quinolino[3,2,1-*kl*]phenothiazin-9-one (denoted QPT-TEG), was a viscose fluid and was miscible with many polar organic solvents (Figure S2).

We examined the electron density distribution around the orthonormal natural atomic orbitals in the QPT core and the redox intermediates QPT^•+^ (upon oxidation) and QPT^•–^ (upon reduction) by NPA, which is an effective way to describe the electron distribution in compounds of high ionic character with improved numerical stability,^47^ and compared it with those of two similar structures, (10-methyl-10*H*-phenothiazin-1-yl)(phenyl)methanone and 1-(2-(10*H*-phenothiazin-10-yl)phenyl)ethan-1-one (denotes PTZ-KT and BP-PT, respectively), without fused conjugation (Figure 1c; the values of NPA charges are provided in Table S1). In comparison with PTZ-KT and BP-PT, QPT-OMe showed a greater NPA charge value in the quaternary N and a lower NPA charge value in the C and O atoms of the ketone, suggesting that the fused conjugation promotes the electron density to redistribute from the electron-rich quaternary N moiety to the electron-deficient ketone moiety. Furthermore, in the cases of both QPT^•+^ and QPT^•–^, the NPA charge values of N, C, and O for both increased upon oxidation, where a large increase was found at the quaternary N, implying that the negative charge transfer from C=O to N^+^ is obstructed. Similarly, the NPA charge values of N, C, and O both decreased upon reduction, suggesting that the positive charge transfer from quaternary N to C–O^–^ is obstructed. Therefore, the oxidation potential of quaternary N is increased and the reduction potential of C=O is decreased in QPT in comparison with the other two structures, resulting in a broadened voltage gap between the two redox moieties. Such a charge transfer was further experimentally confirmed by excluding the influences from the electron-donating quaternary N and electron-withdrawing C=O (Figure S3).

Frontier molecular orbital theory was then used to validate the redox centers on QPT. As is known, for n-type redox centers, a higher energy level of the lowest unoccupied molecular orbital (LUMO) indicates a weaker electron affinity and a lower redox potential. For p-type redox centers, a lower energy level of the highest occupied molecular orbital (HOMO) indicates a stronger electron affinity and a higher redox potential.^42,48^ As is shown in Figure S4, the calculated HOMO energy level of QPT was significantly lower than those of PTZ-KT and BP-PT. It is notable that QPT showed a moderate LUMO energy level that was slightly lower than that of BP-PT, because the benzene ring of BP-PT has a high rotational degree of freedom, which can promote the conjugation integration with the ketone moiety, increasing the LUMO energy level. Nevertheless, QPT exhibited the broadest energy gap that would translate to the largest potential separation of the oxidation and reduction reactions. In addition, the HOMO and LUMO energy levels of QPT and its derivatives (Figure S5) were distributed exclusively on the quaternary N (oxidation reaction) and ketone moieties (reduction reaction), respectively. The same molecular orbital energy levels demonstrated the same characteristics of the three derivatives. Moreover, we calculated the HOMO and LUMO energy levels of other BRMs following the same design strategy (Figure S6) and found that the potential difference between p-type and n-type redox-active moieties can be readily broadened by a fused conjugation. This further validates the rationale of our strategy. In summary, the theoretical investigations confirmed that fused conjugation is a simple yet effective way to increase the voltage gap of BRMs.

The redox kinetics of QPT-OMe was investigated by cyclic voltammetry (CV) using tetrabutylammonium bis(trifluoromethylsulfonyl)imide (TBA-TFSI) in acetonitrile as the electrolyte. As shown in Figure 2a, two pairs of symmetrical cathodic and anodic peaks are clearly observed. The half-wave potentials at −2.04 and 0.72 V (vs Ag^+^/Ag) correspond to the reduction and oxidation reactions of QPT-OMe, respectively. Accordingly, the open-circuit voltage when QPT-OMe is used as the active material in RFBs is estimated to be ca. 2.76 V, which is one of the highest voltage for bipolar molecules found so far.^33,34^ Notably, cathodic and anodic peaks remained highly symmetrical across a wide sweep rate between 5 and 5000 mV s^–1^. A good linear relationship can be found between the peak current and the square root of the sweep rate (Figure 2b), indicating that the redox reactions are diffusion-controlled.^49^ The diffusion coefficient (*D*_0_) of QPT-OMe was calculated to be 2.3 × 10^–6^ cm^2^ s^–1^ by using the Randles–Sevcik equation. The electron-transfer rate constant (*k*_0_) was also calculated according to Nicholson’s analysis,^50^ as shown in Figure 2c. Impressively, QPT-OMe/QPT^•–^-OMe and QPT^•+^-OMe/QPT-OMe redox couples showed electron-transfer rate constants of 1.4 × 10^–2^ and 1.6 × 10^–2^ cm s^–1^, respectively. This is because an outer-sphere one-electron transfer from or to an aromatic π system requires a relatively low energy of reorganization.^39^ Such a high diffusion coefficient and electron-transfer rate constant may favor the achievement of better cell performance, particularly in minimizing the voltage hysteresis.^51^ In addition, we carried out a CV test on a mixture of QPT-OME and QPT-TEG. The waveforms of the two BRMs were overlapped completely (Figure S7), demonstrating that functionalization with polar groups is a readily and effectively accessible method to enhance the energy density, without severely changing the redox behavior and electronic configuration of the QPT core.

**Figure 2:**
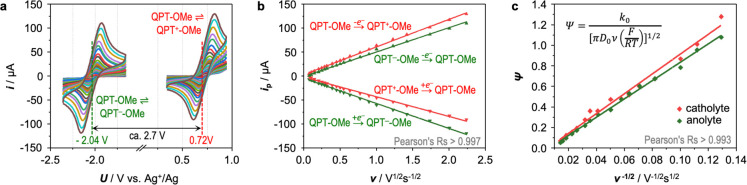
CV study of the redox kinetics of QPT-OMe. (a) CV profiles of an acetonitrile solution with 2 mM QPT-OMe and 100 mM TBA-TFSI at various sweep rate from 5 to 5000 mV s^–1^. (b) Plots of anodic and cathodic peak currents (*i*_p_) versus the square root of the sweep rate (ν^1/2^) derived from the CV profiles. (c) Plots of the kinetic parameter Ψ versus the reciprocal of the square root of the sweep rate (ν^–1/2^). The expression of Ψ is shown in the upper right equation. Ψ is a dimensionless kinetic parameter, which is dependent on ν, the diffusion coefficient (*D*_0_), and the electron-transfer rate constant (*k*_0_) of the electroactive species. *F*, *R*, and *T* represent the Faradaic constant, gas constant, and absolute temperature, respectively.

To verify the feasibility of QPT-OMe as an active material in a symmetrical nonflow cell, an acetonitrile solution containing QPT-OMe and TBA-TFSI was used as both the catholyte and anolyte, and a porous membrane was used as the separator. The cell architecture and electrochemical processes are illustrated in Figure 3a. The cathode and anode compartments of the cell were injected with an equal volumes of 0.1 mL of 0.025 M QPT-OMe in a 0.5 M TBA-TFSI/acetonitrile solution with a theoretical capacity of 335 mAh L^–1^. The typical charge/discharge profiles shown in Figure 3b exhibited distinct charge/discharge plateaus at all measured current densities. At a low current density (<5 mA cm^–2^), the discharge voltage was 2.5–2.7 V with a relatively small voltage hysteresis. To our knowledge, this is one of the highest values for RFBs.^31,36,37,52^ The utilization ratios of QPT-OMe at current densities of 1, 2, 5, 10, and 20 mA cm^–2^ were 77.6%, 86.3%, 97.6%, 94.3%, and 65.4% with the corresponding Coulombic efficiencies (CEs) of 70.0%, 82.1%, 90.6%, 92.9%, and 92.8%, respectively. The internal shuttle effect caused by electrolyte crossover through the porous ion-conductive membrane should be responsible to the “overcharge” phenomenon. The utilization ratio and the corresponding Coulombic efficiency kept increasing with an increase of current density below 5 mA cm^–2^. The reason should be the result of the alleviation of the internal shuttle effect as the charge–discharge time is shortened.^53^ Meanwhile, at a higher current density (>10 mA cm^–2^), the cell showed a more obvious voltage hysteresis due to the concentration polarization that limits the mass transport in the electrolyte and across the separator, causing the utilization ratio and CE to decrease at a current density of 20 mA cm^–2^. Figures 3c,d shows the charge–discharge profiles after different cycles and long-term capacity retention of the symmetrical cell. The voltage plateau remained almost unchanged for 900 cycles. This was further proven by the differential capacity analysis shown on the right side of Figure 3c, where the potential range of charging (2.7–3.1 V) and discharging processes (2.4–2.7 V) as well as peak positions showed no change during the long-term cycling, suggesting that the capacity was solely contributed by the aforementioned redox reactions. The capacity retention was ca. 63.5% after 900 cycles with a decay rate of ca. 0.4‰ per cycle, and the CE and energy efficiency (EE) reached ca. 97% and 84%, respectively. The presented performance is superior to that of most nonaqueous RFBs using either BRMs or asymmetric organic molecules as active materials.^31,36,37,52^

**Figure 3:**
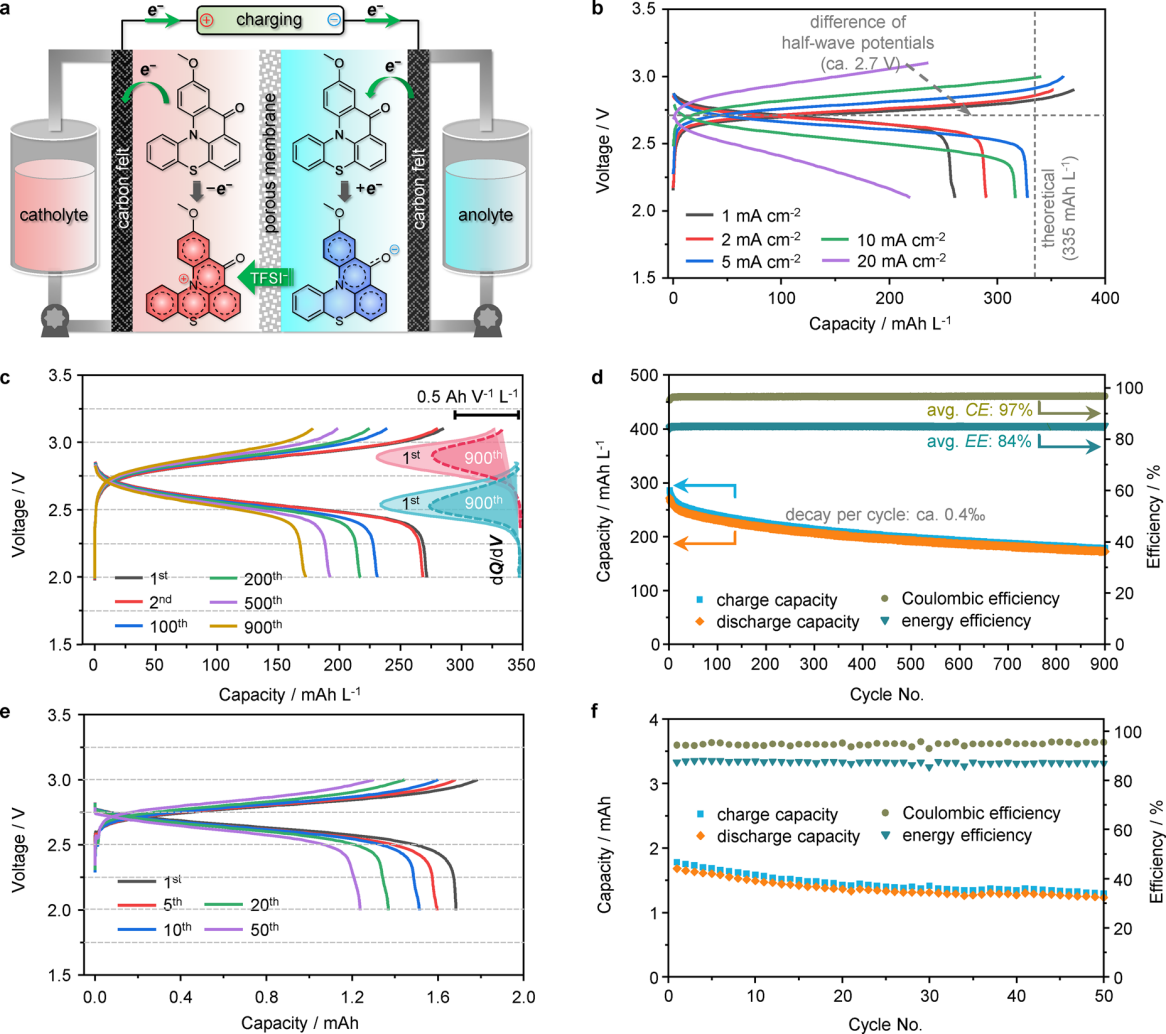
Galvanostatic charge–discharge behavior of a symmetrical RFB based on QPT-OMe. (a) Cell architecture and the electrochemical processes during cell charging. (b) Representative charge–discharge profiles at different current densities. (c) Selected charge–discharge profiles during long-term cycling. (d) Corresponding capacity retention, CE, and EE, of a nonflow cell. The nonflow cell was evaluated at a constant current density of 5 mA cm^–2^ with equal 0.1 mL volumes of 25 mM QPT-OMe and 0.5 M TBA-TFSI in acetonitrile as the electrolyte. (e) Representative charge–discharge profiles during long-term cycling. (f) Corresponding capacity retention, CE, and EE of a flow cell. The flow cell was evaluated at a constant current density of 10 mA cm^–2^ with equal 3 mL volumes of 25 mM QPT-OMe and 0.5 M TBA-TFSI in acetonitrile as the electrolyte.

We further tested the electrochemical performance of QPT-OMe in a flow cell (Figure S8). This cell exhibited charge/discharge profiles similar to those of the nonflow cell, shown in Figure 3e with a utilization ratio of QPT-OMe of ca. 83.5% and a capacity retention of 73.8% after 50 cycles. The corresponding CE and EE reached 95% and 87%, respectively, as demonstrated in Figure 3f. The satisfactory structural stability of QPT-OMe could be further demonstrated by the almost identical CV profiles of the leachates of the anolyte and catholyte after cycling (Figure S9). The capacity decay may arise from unpredictable side reactions, such as that of a carbonyl-based radical with acetonitrile through nucleophilic substitution,^54,55^ increased internal resistance (Figure S10) caused by the surface oxidation of carbon felt at a high oxidation potential (1.4 V vs NHE), and a possible precipitation and deactivation of trapped active materials in the porous graphite plate as a result of solvent evaporation.^56,57^ Moreover, a symmetric nonflow cell with a higher concentration was examined by using QPT-TEG due to its higher solubility in comparison to QPT-OMe. Figure S11 shows the charge/discharge profiles at different cycles, where the voltage plateaus were maintained at ca. 2.3 V upon discharging. The utilization rate of the active material in the first cycle was 74.6%, and the discharge capacity remained at 63% after 200 cycles (Figure S12). In comparison to the cell with a low concentration, both the active material utilization and capacity retention decreased, which is a common phenomenon in RFBs using a concentrated electrolyte. This is because both the electrolyte viscosity and mass transfer polarization increased with an increase in the active material concentration.^58,59^ In addition, we performed CV (Figure S13) and cycling tests (Figure S14) of the QPT-TEG with different solvents. On the whole, QPT-based molecules exhibited the best performance when acetonitrile was used as the electrolyte solvent.

The capability of QPT-OMe as a BRM was further confirmed in a pole reversal experiment with a nonflow cell, where the polarity of the cell was reversed every 50 cycles for 200 cycles, followed by another 300 cycles without being reversed. The charge/discharge profiles (Figure 4a,b) exhibited a high symmetry throughout the entire cycling process, and no deviations could be observed between two consecutive periods of charging or discharging. The capacity retention reached ca. 56% after 500 cycles, and the CE and EE remained at high levels of ca. 94% and 86%, respectively, as indicated in Figure 4c. It is worth noting that the charge and discharge capacities increased initially after a pole switch and were stabilized after a few cycles. This is ascribed to the fact that an additional capacity was sacrificed to balance the remained QPT^•+^-OMe and QPT^•–^-OMe in the prior cycle in the catholyte and anolyte, respectively, resulting in a sudden drop of both CE and EE. As the cycling continued, the rebalancing of the whole battery system by continued pole reversals would alleviate such a capacity increase and reach a steady state. The results shown in Figures 3 and 4 demonstrated the good stability and chemical reversibility of QPT-OMe. In comparison with previously reported BRMs (summarized in Table S2), QPT-OMe delivered not only one of the highest output voltages (2.5 V) so far but also a low molecular weight, which led to an energy density of 202 W h kg^–1^ in comparison with those of typically less than 150 W h kg^–1^.^8,16,17,39,60−64^

**Figure 4:**
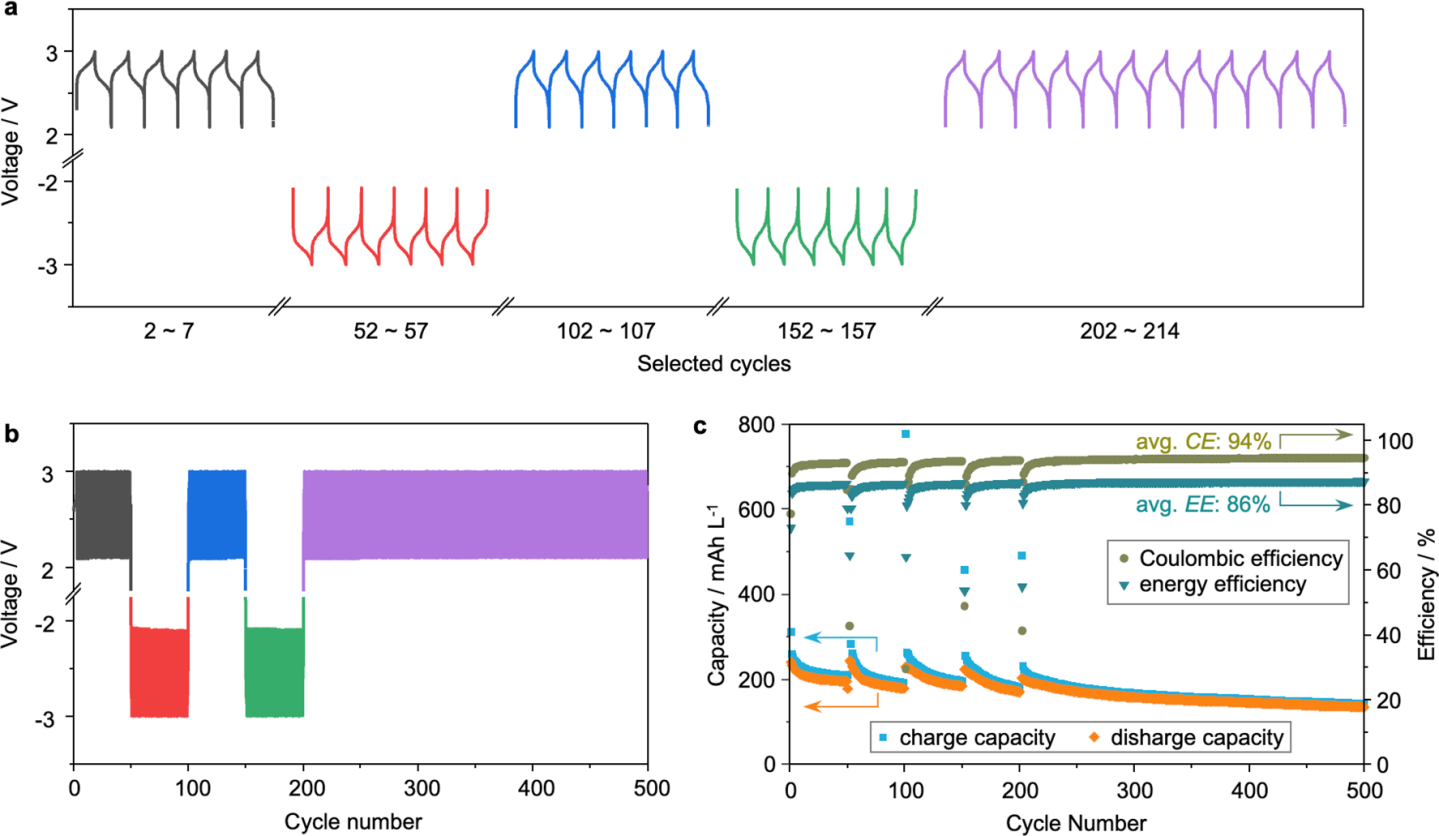
Pole reversal experiment of QPT-OMe in a symmetrical cell. (a) Representative galvanostatic charge/discharge profiles and (b) long-term battery cycling of the polarity reversal experiment. (c) Corresponding charge/discharge capacity, CE, and EE. The nonflow cell was evaluated at a current density of 5 mA cm^–2^ with equal 0.1 mL volumes of 25 mM QPT-OMe and 0.5 M TBA-TFSI in acetonitrile as the electrolyte. The polarity of the cell was reversed 4 times after each 50 cycles, and then cycling was continued for another 300 times.

Furthermore, three-electrode spectroelectrochemical X-band electron paramagnetic resonance (EPR)^65^ and UV–vis spectroscopy were used to detect the redox intermediates during the redox reactions of QPT-OMe. The electrochemical EPR spectra shown in Figure 5a indicated radical signals at both the oxidized and reduced states of QPT-OMe, and the spectra match well with simulated spectra.^66,67^ At the oxidized state, the spectrum exhibited three peaks with strong hyperfine coupling (*a* ≈ 0.65 mT). This is attributed to the localized radical electron that is distributed mainly around the N and S atoms in the phenothiazine moiety and extends to the entire conjugated structure, as revealed by the spin density distribution shown in Figure 5b. At the reduced state, the spectrum showed five peaks with an integration ratio of approximately 1:4:6:4:1 and a hyperfine coupling constant (*a* ≈ 0.36 mT), which is due to the four spin resonance H atoms linking with the four carbon atoms shown in turquoise (Figure 5c). Different from QPT^•+^-OMe, the radical electron in QPT^•–^-OMe is distributed mainly in the conjugated plane of the acridone skeleton, according to the spin density distribution simulation of radical ions (Figure 5b,c). The extended conjugation, particularly for QPT^•–^-OMe, might account for enhancement of the stability of ketone radicals that usually require inert-gas protection during cell operation to avoid nucleophilic attack and/or aeration-induced degradation by the coexisting species in the electrolyte or need a complicated molecular engineering method to protect the reactive center via steric hindrance and the like.^68^

**Figure 5:**
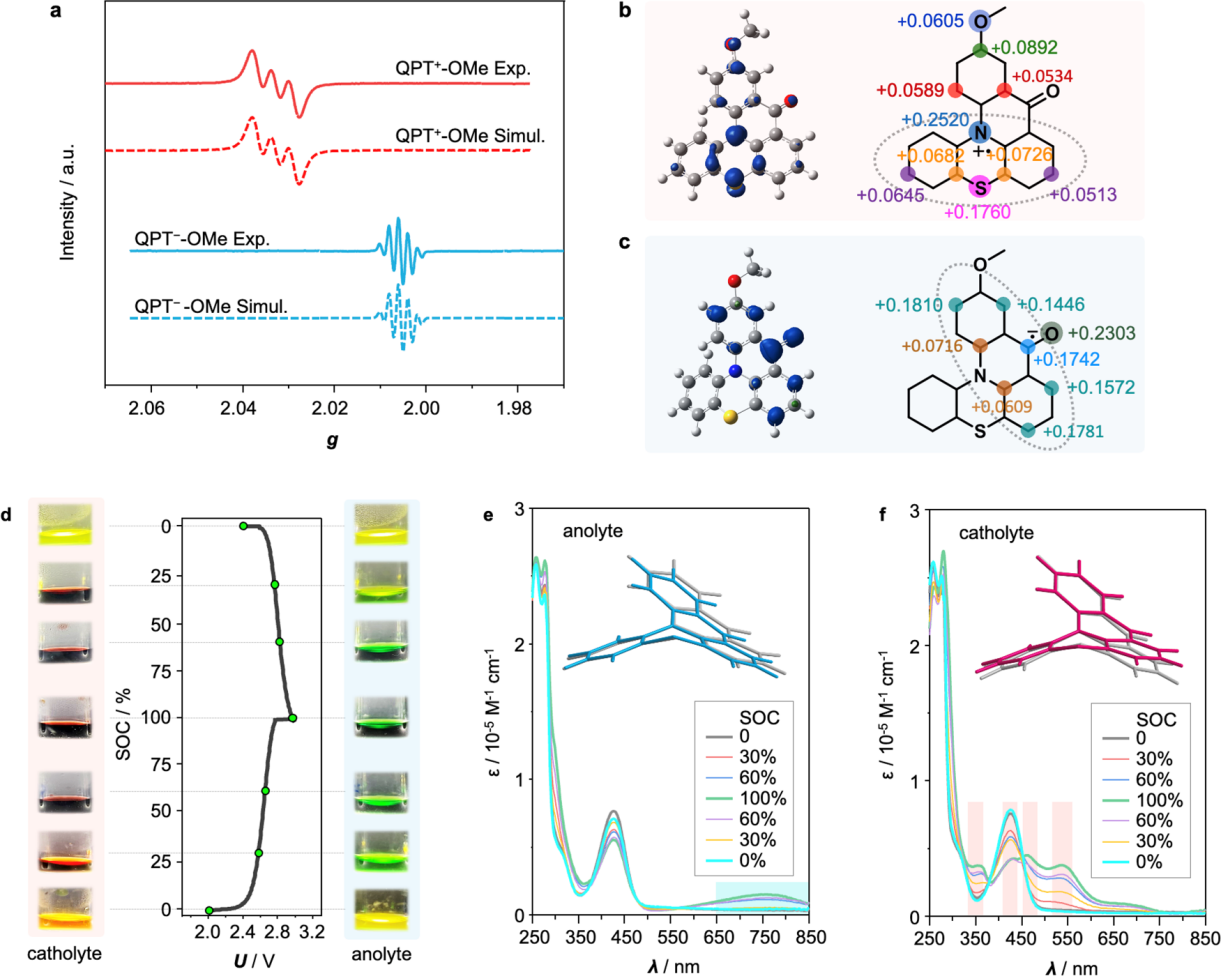
Spectroscopic study on the structural variation of QPT-OMe during the electrochemical processes. (a) Experimental and simulated EPR spectra of QPT^•+^-OMe and QPT^•–^-OMe; Spin density distribution of (b) QPT^•+^-OMe and (c) QPT^•–^-OMe. The panels on the left represent the visualized spin density that is in proportion to the distribution size, while the panels on the right give the calculated values on different atoms. (d) Digital images showing the regular color change of the anolyte and catholyte at different SOCs of 0%, 30%, 60%, 100%, 60%, 30%, and 0% in a complete charge–discharge cycle. (e, f) Corresponding UV–vis spectra at different SOCs. The molecular structures in the insets indicate the structural distortion with respect to QPT-OMe sketched in gray.

The digital images shown in Figure 5d reveal that both the anolyte and catholyte showed a strong and periodic color change during the oxidation and reduction processes. The color change can be directly used to monitor the state of charge of a cell, avoiding the use of a costly and complex battery management system.^69^ As shown in Figure 5e, the UV–vis spectra of the anolyte showed no obvious change at the wavelength region below 500 nm during a charge/discharge cycle. A broad adsorption band gradually appeared at 650–850 nm when the state of charge (SOC) increased from 0% to 100%. For the catholyte, the absorptivity around 425 nm slightly decreased when the SOC increased, while the intensities of the bands at ca. 350, 470, and 540 nm increased (Figure 5f). In addition, reversible spectroscopic changes were observed during cycling that indicated a decent stability and reversibility of the electrochemical process. As is known, new adsorption bands appear when the energy difference between π and π* orbitals of a conjugated structure decreases upon gaining additional negative or positive charge.^70,71^ The appearance of new adsorption bands toward longer wavelength in Figure 5e is expected to be correlated with the structure distortion of QPT upon oxidation. The optimized structure of QPT^•–^ during the reduction process indicated a small distortion that mainly occurs on the acridone skeleton plane (inset of Figure 5e), whereas a more obvious distortion was found in the entire conjugated structure during the oxidation process, and the phenothiazine moiety of QPT^•+^ tends to be more planar and thus have a better conjugation (inset of Figure 5f). The variations in the conjugation of QPT^•+^ and QPT^•–^ thus result in distinctly different UV–vis adsorption. In addition, a nucleus-independent chemical shift (NICS) analysis (Figure S16) confirmed that the change in NICS values caused by structural distortion is mainly found in the heterocycle and the two attached hexatomic rings in QPT-OMe and is more likely to be delocalized throughout the entire conjugation in QPT^•+^-OMe. On the basis of the above analysis, the extended conjugation in the redox intermediates of QPT-OMe is advantageous in maintaining chemical stability in comparison with individual quaternary N or ketone redox centers, suggesting the fused conjugation strategy not only provides a platform to develop new BRMs with broadened voltage gaps but also endows the as-designed BRMs with enhanced stability in comparison with those using individual redox-active moieties. Further enhancement in solubility and voltage would occur in turn by modifying the general formula with more soluble motifs and integrating other redox centers with a maximized redox potential.

In conclusion, a general strategy is proposed to develop new small bipolar molecules for symmetric RFBs to alleviate the major issue of electrolyte crossover. The strategy uses fused conjugation to regulate the electron density redistribution and further to regulate the redox potentials of the integrated redox-active moieties. A new bipolar molecule, QPT-OMe, based on a fused conjugation of quaternary N and ketone redox moieties is designed and used as the active material in a symmetrical cell. The results showed that the fused conjugation can promote the electron density in the conjugation to redistribute from the electron-deficient ketone moiety to the electron-rich quaternary N moiety, resulting in the broadening of the voltage gap between the two redox moieties. The molecular geometry and a spectroscopic analysis of the redox intermediates revealed that the negative charge on the reduced QPT-OMe is mainly delocalized in the heterocycle and the two attached hexatomic rings, while the positive charge on the oxidized QPT-OMe is more likely to be delocalized throughout the entire conjugation. In an application as the sole active material in a symmetrical cell, QPT-OMe delivered a cell voltage of 2.5 V, which is one of the highest cell voltages achieved in BRMs for symmetric RFBs so far, and maintained fast redox kinetics and decent cycling stability. The strategy proposed in this study may contribute to designing small-molecular-weight BRMs that are essential to overcome the issues of electrolyte crossover and low cell voltage of RFBs, leading to enriched cell chemistry and simplified cell architecture.

## Supporting Information Available

Details of experimental procedures and methods, synthetic routes, DFT simulation results, summary of reported redox-active materials in flow batteries, and additional discussion, characterization data, and electrochemical analysis results (PDF)
