# Supplementary material for: Effective Design Strategy of Small Bipolar Molecules through Fused Conjugation toward 2.5 V Based Redox Flow Batteries
Source: ACS Energy Lett. 2022 Mar 8;7(4):1274–83. doi: 10.1021/acsenergylett.2c00198 (PMC9097584; doi:10.1021/acsenergylett.2c00198)
Supplement: Supplementary file 1 — nz2c00198_si_001.pdf [file nz2c00198_si_001.pdf]

Supporting Information

An effective design strategy of small bipolar molecules through fused conjugation towards 2.5 V based redox flow batteries

Yue Liu,[a,b,+] Gaole Dai,[c,+] Yuanyuan Chen,[a] Ru Wang,[a] Huamei Li,[a] Xueliang Shi,[d] Xiaohong Zhang,*[a] Yang Xu*[b] and Yu Zhao*[a,c]

[a] Institute of Functional Nano & Soft Materials (FUNSOM), Jiangsu Key Laboratory for Carbon-based Functional Materials & Devices, Soochow University, 199 Renai Road, Suzhou, Jiangsu 215123, P.R. China, E-mail: xiaohong_zhang@suda.edu.cn

[b] Department of Chemistry, University College London, 20 Gordon Street, London, WC1H 0AJ, UK, E-mail: y.xu.1@ucl.ac.uk

[c] College of Materials, Chemistry and Chemical Engineering, Hangzhou Normal University, 2318 Yuhangtang Road, Hangzhou, Zhejiang 311121, P.R. China, E-mail: yuzhao@hznu.edu.cn

[d] Shanghai Key Laboratory of Green Chemistry and Chemical Processes, School of Chemistry and Molecular Engineering, East China Normal University, 500 Dongchuan Road, Shanghai 200062, P.R. China

[+] These authors contribute equally.

Table of Contents

Experimental Procedures ········· 2

Figure S1 ········· 11

Figure S2 ········· 11

Table S1 ········· 12

Figure S3 ········· 17

Figure S4 ········· 18

Figure S5 ········· 18

Figure S6 ········· 19

Figure S7 ········· 19

Figure S8 ········· 20

Figure S9 ········· 20

Figure S10 ········· 21

Figure S11 ········· 21

Figure S12 ········· 22

Figure S13 ········· 22

Figure S14 ········· 23

Figure S15 ········· 24

Figure S16 ········· 24

Table S2 ········· 25

NMR spectrum of QPT-OMe and QPT-TEG········· 27-30

References ········· 31

Experimental Procedures

Materials and general characterization method

All reagents were purchased from commercial sources without further purification. ^{1}H and ^{13}C NMR spectra were recorded using Advance 500 MHz Bruker spectrometer with tetramethylsilane (TMS) as the internal standard. The chemical shift was recorded in ppm and the following abbreviations were used to explain the multiplicities: s = singlet, d = doublet, t = triplet, m = multiplet, br = broad. MALDI-TOF mass spectra were recorded on a Bruker Ultraflextreme instrument. UV spectra was recorded on a PerkinElmer UV-Vis spectrophotometer. Column chromatography was performed on silica gel (200-300 mesh). CW X-band EPR spectra for radicals were acquired on Bruker EMX instrument EMXPLUS-10/12.

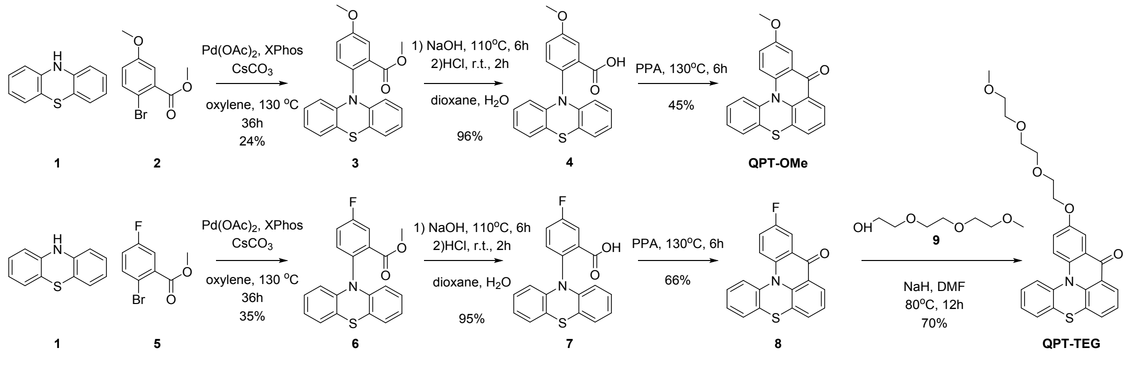

Scheme S1. Preparation routes to QPT-OMe and QPT-TEG.

Synthesis of methyl 5-methoxy-2-(10H-phenothiazin-10-yl)benzoate (3)

A mixture of 10*H*-phenothiazine (**1**) (0.796 g, 4.0 mmol), methyl 2-bromo-5-methoxybenzoate (**2**) (1.029 g, 4.2 mmol), Cs_2CO_3 (3.910 g, 12.0 mmol), $Pd(OAc)_2$ (90 mg), and XPhos (381 mg) in o-xylene (40 mL) was heated at 130 °C for 36 hours after degassing by freeze–pump–thaw and filling in with nitrogen. After the heating was

completed, the mixture was cooled down to room temperature and extracted with dichloromethane (DCM). The organic phase was collected and concentrated. Then, the crude product was purified by column chromatography on silica gel to afford a primrose yellow solid in a yield of 24%. ^{1}H NMR (400 MHz, DMSO-d_6, ppm) δ = 7.57 (d, J = 2.8 Hz, 1H), 7.51 – 7.40 (m, 2H), 6.98 (dd, J = 7.5, 1.7 Hz, 2H), 6.85 (td, J = 7.8, 1.7 Hz, 2H), 6.78 (td, J = 7.4, 1.3 Hz, 2H), 5.95 (dd, J = 8.2, 1.3 Hz, 2H), 3.91 (s, 3H), 3.65 (s, 3H). ^{13}C NMR (101 MHz, DMSO-d_6, ppm) δ = 165.11, 158.91, 143.25, 134.57, 132.50, 131.27, 127.11, 126.26, 122.21, 120.13, 118.13, 115.07, 55.80, 52.33. MALDI-TOF MS: $C_{21}H_{17}NO_3S$, exact mass: 363.093, found: 363.080.

Synthesis of 5-methoxy-2-(10H-phenothiazin-10-yl)benzoic acid (4)

A mixture of methyl 5-methoxy-2-(10*H*-phenothiazin-10-yl)benzoate (**3**) (0.544 g, 1.5mmol), NaOH (0.480 g, 12 mmol), H_2O (10 mL) and dioxane (20 mL) was heated at 110 ^{o}C for 6 hours under nitrogen atmosphere. After cooling to room temperature, an aqueous hydrogen chloride (1 M, 10-20 mL) was added dropwise in the mixture until a white solid precipitated and pH < 1. After stirring 2 hours, the mixture was filtered, washed completely with water and dry in vacuum to give the pure product in 96% yield. ^{1}H NMR (400 MHz, DMSO-d_6, ppm) δ = 13.00 (s, 1H), 7.57 (dd, J = 2.4, 1.0 Hz, 1H), 7.43 – 7.33 (m, 2H), 6.93 (dd, J = 7.5, 1.6 Hz, 2H), 6.82 (ddd, J = 8.2, 7.3, 1.7 Hz, 2H), 6.74 (td, J = 7.4, 1.3 Hz, 2H), 5.89 (dd, J = 8.2, 1.3 Hz, 2H), 3.89 (s, 3H). ^{13}C NMR (101 MHz, DMSO-d_6, ppm) δ = 166.06, 158.87, 143.22, 133.99, 133.95, 131.29, 127.03, 126.07, 121.94, 120.09, 117.72, 116.85, 115.14, 55.71. MALDI-TOF MS: $C_{20}H_{15}NO_3S$, exact mass: 349.077, found: 349.054.

Synthesis of 11-methoxy-9H-quinolino[3,2,1-kl]phenothiazin-9-one (QPT-OMe)

A mixture of 5-methoxy-2-(10*H*-phenothiazin-10-yl)benzoic (**4**) acid (0.349 g, 1.0mmol) and polyphosphoric acid (PPA) (50 mL) was heated at 130 °C for 6 hours under nitrogen atmosphere. After cooling down to room temperature, the reaction mixture was added dropwise with ice water (200 mL) and extracted with DCM. The organic phase was collected, dried by anhydrous $MgSO_4$ and concentrated. The crude product was purified by column chromatography on silica gel to afford a yellow solid in a yield of 45%.^{1}H NMR (400 MHz, DMSO-d_6, ppm) δ = 8.06 (dd, J = 8.0, 1.4 Hz, 1H), 7.82 (d, J = 9.2 Hz, 1H), 7.73 (dt, J = 7.6, 1.3 Hz, 1H), 7.66 (d, J = 3.1 Hz, 1H), 7.58 – 7.49 (m, 1H), 7.47 – 7.36 (m, 2H), 7.35 – 7.23 (m, 3H), 3.92 (s, 3H).^{13}C NMR (101 MHz, DMSO-d_6, ppm) δ = 177.14, 155.88, 142.11, 139.00, 135.11, 130.93, 129.03, 128.08, 126.40, 125.97, 125.58, 124.70, 124.23, 123.56, 122.93, 122.75, 122.72, 121.60, 106.17, 55.61. MALDI-TOF MS: $C_{20}H_{13}NO_2S$, exact mass: 331.067, found: 331.078.

Synthesis of methyl 5-fluoro-2-(10H-phenothiazin-10-yl)benzoate (6)

A mixture of 10*H*-phenothiazine (**1**) (0.796 g, 4.0 mmol), methyl 2-bromo-5-fluorobenzoate (**5**) (0.979 g, 4.2 mmol), cesium carbonate (3.910 g, 12.0 mmol), catalyst $Pd(OAc)_2$ (90 mg), and ligand 2-dicyclohexylphosphino-2;-biphenyl]-2-yl]phosphane (XPhos) (381 mg) in o-xylene (40 mL) was degassed by freeze–pump–thaw and then filled in with nitrogen. The mixture was heated for 36 hours at 130 °C and extracted with DCM after cooling to room temperature. The blended organic phase was collected and concentrated to afford crude product. Then, the crude product was

purified by column chromatography on silica gel to afford a yellow solid in a yield of 35%. ^{1}H NMR (400 MHz, DMSO-d_6, ppm) δ = 7.89 (dd, *J* = 8.9, 3.1 Hz, 1H), 7.76 (td, *J* = 8.4, 3.1 Hz, 1H), 7.66 (dd, *J* = 8.7, 5.2 Hz, 1H), 7.00 (dd, *J* = 7.4, 1.7 Hz, 2H), 6.86 (td, *J* = 7.8, 1.7 Hz, 2H), 6.80 (td, *J* = 7.4, 1.3 Hz, 2H), 5.94 (dd, *J* = 8.2, 1.3 Hz, 2H), 3.66 (s, 3H). ^{13}C NMR (101 MHz, DMSO-d_6, ppm) δ = 164.23, 164.20, 162.40, 159.94, 142.86, 136.01, 135.92, 135.12, 135.08, 133.75, 133.67, 127.17, 126.37, 122.45, 121.89, 121.67, 119.10, 118.85, 118.30, 115.12, 52.56. MALDI-TOF MS: $C_{20}H_{14}FNO_2S$, exact mass: 351.073, found: 351.021.

Synthesis of 5-fluoro-2-(10H-phenothiazin-10-yl)benzoic acid (7)

A mixture of methyl 5-fluoro-2-(10*H*-phenothiazin-10-yl)benzoate (**6**) (0.702 g, 2 mmol), NaOH (0.640 g, 12 mmol), H_2O (10 mL) and dioxane (20 mL) was heated at 110 ^{o}C for 6 hours under nitrogen atmosphere. After cooling to room temperature, an aqueous hydrogen chloride (1M, 10-20 mL) was added dropwise in the mixture until a white solid precipitated and pH < 1. After stirring 2 hours, the mixture was filtered, washed with water completely and dry in vacuum to give the pure product in 95% yield. ^{1}H NMR (400 MHz, DMSO-d_6, ppm) δ = 13.28 (s, 1H), 7.87 (dd, *J* = 9.0, 3.1 Hz, 1H), 7.71 (td, *J* = 8.3, 3.1 Hz, 1H), 7.56 (dd, *J* = 8.7, 5.2 Hz, 1H), 6.96 (dd, *J* = 7.5, 1.6 Hz, 2H), 6.84 (td, *J* = 7.8, 1.7 Hz, 2H), 6.77 (td, *J* = 7.4, 1.2 Hz, 2H), 5.89 (dd, *J* = 8.2, 1.1 Hz, 2H). ^{13}C NMR (101 MHz, DMSO-d_6, ppm) δ = 165.18, 165.16, 162.44, 159.98, 142.85, 135.43, 135.35, 135.27, 135.07, 135.04, 127.10, 126.18, 122.19, 121.80, 121.58, 119.06, 118.82, 117.87, 115.15. MALDI-TOF MS: $C_{19}H_{12}FNO_2S$, exact mass: 337.057, found: 337.260.

Synthesis of 11-fluoro-9H-quinolino[3,2,1-kl]phenothiazin-9-one (8)

A mixture of 5-fluoro-2-(10*H*-phenothiazin-10-yl)benzoic acid (**7**) (0.505 g, 1.5 mmol) and PPA (50 mL) was heated at 130 °C for 6 hours under nitrogen atmosphere. After cooling down to room temperature, the reaction mixture was added dropwise with ice water (200 mL) and extracted with DCM. The organic phase was collected, dried by anhydrous $MgSO_4$ and concentrated. Then, the crude product was purified by column chromatography on silica gel to afford a yellow solid in a yield of 66%.^{1}H NMR (400 MHz, DMSO-d_6, ppm) δ = 8.05 (dd, J = 8.0, 1.4 Hz, 1H), 7.98 – 7.89 (m, 2H), 7.76 (dd, J = 7.5, 1.4 Hz, 1H), 7.66 (ddd, J = 9.3, 8.0, 3.2 Hz, 1H), 7.59 – 7.53 (m, 1H), 7.48 – 7.41 (m, 1H), 7.34 – 7.27 (m, 3H). ^{13}C NMR (101 MHz, DMSO-d_6, ppm) δ = 177.37, 177.34, 160.08, 157.66, 142.69, 139.26, 138.13, 131.81, 129.54, 128.61, 127.15, 126.76, 126.70, 126.30, 125.50, 124.66, 124.26, 124.18, 123.47, 122.28, 122.23, 121.98, 111.31, 111.08. MALDI-TOF MS: $C_{19}H_{10}FNOS$, exact mass: 319.047, found: 319.069.

Synthesis of 11-(2-(2-(2-methoxyethoxy)ethoxy)ethoxy)-9H-quinolino[3,2,1-kl]phenothiazine 9-one (QPT-TEG)

A mixture of 11-fluoro-9*H*-quinolino[3,2,1-*kl*]phenothiazin-9-one (**8**) (0.319 g, 1.0mmol), 2-(2-(2-methoxyethoxy)ethoxy)ethan-1-ol (**9**) (0.164 g, 1.0mmol) and NaH (0.191 g, 5 mmol, wt 60%) in N,N-dimethylformamide (DMF) was heated at 80 °C for 12 hours under nitrogen atmosphere. After cooling to room temperature, the mixture was extracted with DCM. The organic phase was collected, dried by anhydrous $MgSO_4$ and concentrated. The crude product was purified by column chromatography on silica

gel to afford an orange colloidal liquid in a yield of 70%. ^{1}H NMR (400 MHz, DMSO-d_6, ppm) δ = 8.06 (dd, J = 8.0, 1.4 Hz, 1H), 7.82 (d, J = 9.2 Hz, 1H), 7.74 (dd, J = 7.4, 1.4 Hz, 1H), 7.67 (d, J = 3.0 Hz, 1H), 7.57 – 7.52 (m, 1H), 7.45 – 7.40 (m, 2H), 7.32 – 7.25 (m, 3H), 4.29 – 4.24 (m, 2H), 3.84 – 3.80 (m, 2H), 3.62 (dd, J = 5.7, 3.1 Hz, 2H), 3.58 – 3.50 (m, 4H), 3.42 (dd, J = 5.8, 3.7 Hz, 2H), 3.23 (s, 3H). ^{13}C NMR (101 MHz, DMSO-d_6, ppm) δ = 177.11, 155.08, 142.06, 138.95, 135.08, 130.86, 129.00, 128.01, 126.37, 125.93, 125.60, 124.65, 124.22, 123.53, 123.01, 122.92, 122.64, 121.52, 107.00, 71.31, 70.02, 69.86, 69.66, 68.89, 67.75, 58.07, 39.73, 39.52, 39.31. MALDI-TOF MS: $C_{19}H_{12}FNO_2S$, exact mass: 463.145, found: 463.096.

Electrochemical characterizations

Galvanostatic tests were performed on a battery testing system (BT-2043, Arbin instruments). Cyclic voltammetry (CV) studies were performed on an electrochemical workstation (CHI-760E, CH Instruments, Inc.). The three-electrode system was employed for the CV test. Gold disk electrode, platinum wire electrode, and Ag/Ag^+(0.01 M $AgNO_3$ and 0.1 M TBA-TFSI in acetonitrile) electrode were served as the working, the counter, and the reference electrode, respectively.

Diffusion coefficient (D_0) and Electron transfer rate constants (k_0) measurement

Diffusion coefficient (D_0) was calculated by Randles–Sevcik equation [1-3] (Eq. (1)).

$$i_p = 0.4463nFAC\left(\frac{nFvD}{RT}\right)^{1/2} \qquad (1)$$

i_p is current maximum in amps, A is electrode area in cm^2, C is concentration in $mol \cdot cm^{-3}$. n is the number of electrons involving the redox process (n = 1), $D(D_0)$, F, R, T, and

v represent the diffusion coefficient ($cm^2 \cdot s^{-1}$), Faradic constant ($C \cdot mol^{-1}$), gas constant ($J \cdot K^{-1}$ mol^{-1}), absolute temperature (K), and scan rate ($V \cdot s^{-1}$) respectively

The ΔE_p was obtained from reductive and oxidative peak potential gap. Ψ is a dimensionless kinetic parameter, which is calculated from Eq. (2). And then, k_0 was calculated using the slope of the Ψ and $v^{-1/2}$ plot based on the relationship between Ψ and $v^{-1/2}$ (Eq. (3).

$$\Psi = (-0.6288 + 0.0021\Delta E_p)/(1-0.017\Delta E_p) \quad (2)$$

$$\Psi = k_0[\pi DnF/RT]^{-1/2} v^{-1/2} \quad (3)$$

n is the number of electrons involving the redox process (n = 1), $D, F, R, T,$ and v represent the diffusion coefficient ($cm^2 \cdot s^{-1}$), Faradic constant ($C \cdot mol^{-1}$), gas constant ($J \cdot K^{-1}$ mol^{-1}), absolute temperature (K), and scan rate ($V \cdot s^{-1}$) respectively.

Cell studies

The non-flow cell was assembled by using two quartz shells (8 mm in inner diameter, 2.5 mm in height) as the cathode and anode compartments. Each compartment was inserted with a piece of carbon felt as the current collector (8 mm in diameter, 4 mm in thickness) and sealed using a piece of Ti foil (0.5 mm in thickness). A piece of Daramic 175® membrane was used as the separator. The quartz shells, Ti foils, and the membrane were sealed together with Surlyn® resin (Solaronix Meltonix 1170-25). The electrolyte was injected into the quartz shells through the hole on the Ti foil, and sealed with polyimide tape. The flow cell was comprised of a stainless-steel plate (400 × 400 mm, 5 mm in thickness), poly(tetrafluoroethylene) (PTFE) frame (400 × 400 mm, 10 mm in thickness), a PTFE gasket (400 × 400 mm, 0.5 mm in thickness), graphite plates (400

× 400 mm, 5 mm in thickness), a carbon felt (15 mm in diameter, 4 mm in thickness), and a Daramic 175® membrane. Therefore, the component sequence of the flow cell is stainless-steel plate - PTFE frame - PTFE gasket - graphite plate - PTFE frame - carbon felt - PTFE gasket - Daramic 175® membrane - PTFE gasket - graphite felt - PTFE frame - graphite plate - PTFE gasket - PTFE frame - stainless-steel plate. Two pumps were used to circulate an equal volume of QPT-OMe catholyte and anolyte (3 mL, 25 mM QPT-OMe and 0.5 M TBA-TFSI in ACN) into the cell at a fixed flow rate of 20 mL min^{-1}. The polarity of cell was determined by the charge and discharge current direction. All the cells were assembled and tested with a potentiostat (BT-2043, Arbin instruments) in the glovebox (H_2O and O_2 concentration < 1 ppm) at room temperature.

Spectroelectrochemical EPR

The temporal three-electrode spectroelectrochemical X-band electron paramagnetic resonance (EPR) was conducted under the ambient condition using a customized EPR electrochemical cell (as shown in Figure S17) and Bruker EMX instrument (EMXPLUS-10/12). The electrolyte solution of QPT-OMe was prepared and transferred to electrochemical cell under the ambient condition and without degassing. The three-electrodes were added and closed the cell. Gold electrode was served as the working electrode, and the two platinum wire electrodes were served as the counter and the reference electrode, respectively. Then, the electrochemical cell was put into the resonant cavity of the EPR instrument and the bottom part of the EPR electrochemical cell was the detection area (around 5 mm in length). After oxidizing at 1.4 V or reducing at -1.1 V for 60 s, the redox potential was removed and collected the EPR signals.

Calculation method

All DFT calculations were based on Gaussian09 program [4]. RB3LYP/6-31G(d) was used for neutral state structure optimization, UB3LYP/6-31G(d) was used for oxidation and reduction state structure optimization, all geometry optimizations are carried out in vacuum and frequency analysis is performed to ensure that no imaginary frequency occurs. Pop = NPA was used for NPA atomic charge calculation. NPA atomic charge visualization and spin density distribution were used Multifunctional wavefunction analyzer (Multiwfn) [5], Visual molecular dynamics (VMD) [6] and GaussView [4].

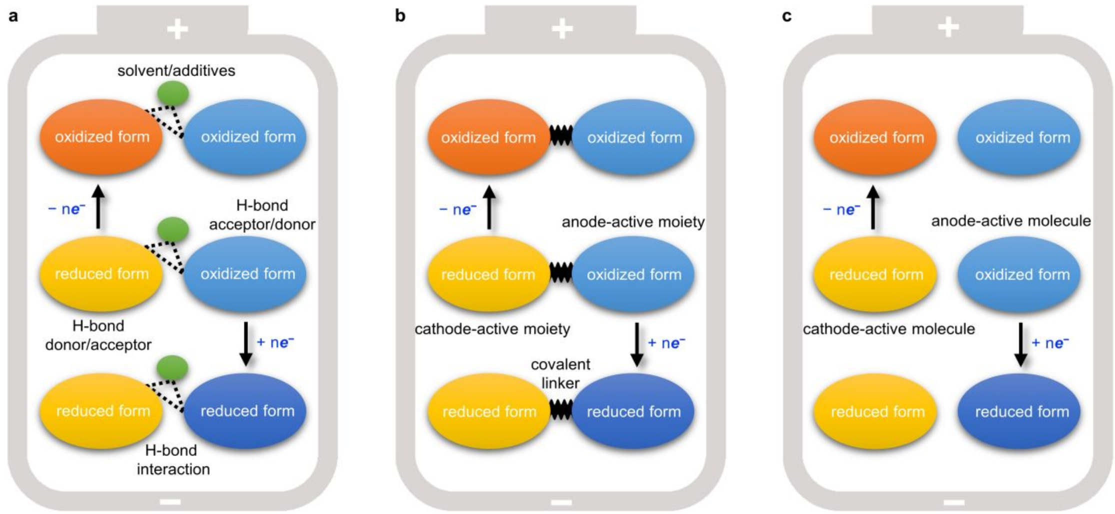

Figure S1. Strategies developed to construct SRFBs and the transformation of "artificial bipolar molecules" during charging in the electrolyte: a) formation of bipolar eutectic mixtures; b) combination of different redox-active moieties through covalent bonding; c) physically mixed anode- and cathode-active molecules.

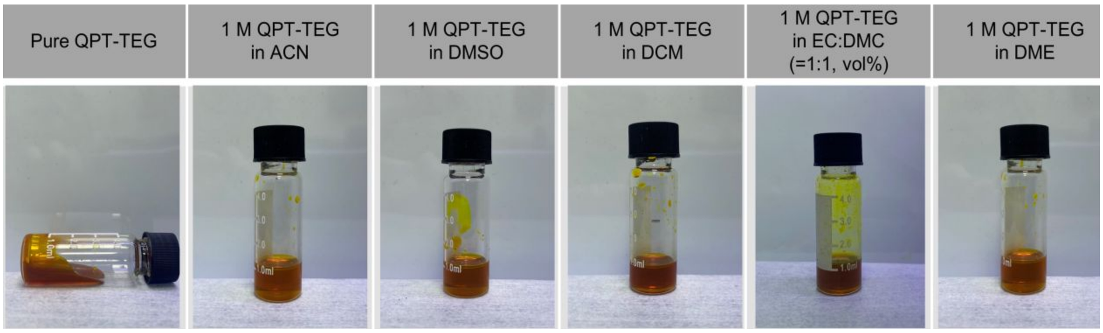

Figure S2. Digital images of pure QPT-TEG and 1 M QPT-TEG in different solvents. The solvents used are acetonitrile (ACN), dimethyl sulfoxide (DMSO), dichloromethane (DCM), ethylene carbonate/diethyl carbonate (EC: DMC = 1:1, vol.%), and dimethoxyethane (DME), respectively.

Table S1. NPA charge values of PTZ-KT, BP-PT, QPT, $QPT^{\bullet+}$, and $QPT^{\bullet-}$.

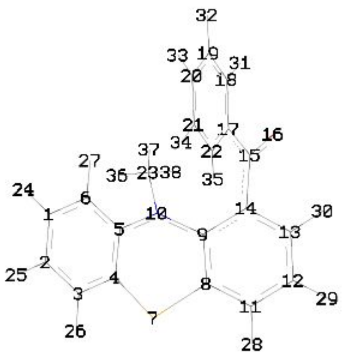

PTZ-KT

No.	Atom	Charge	No.	Atom	Charge	No.	Atom	Charge
1	C	-0.226	14	C	-0.260	27	H	0.240
2	C	-0.248	15	C	0.184	28	H	0.244
3	C	-0.231	16	C	-0.157	29	H	0.237
4	C	-0.218	17	C	-0.201	30	H	0.236
5	C	0.179	18	C	-0.229	31	H	0.237
6	C	-0.252	19	C	-0.205	32	H	0.241
7	S	0.333	20	C	-0.213	33	H	0.244
8	C	-0.218	21	C	0.563	34	H	0.245
9	C	0.178	22	O	-0.522	35	H	0.258
10	N	-0.443	23	C	-0.770	36	H	0.249
11	C	-0.230	24	H	0.236	37	H	0.246
12	C	-0.249	25	H	0.237	38	H	0.265
13	C	-0.225	26	H	0.243			

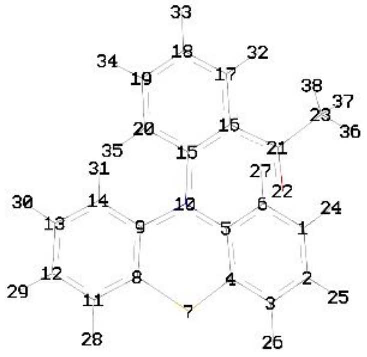

BP-PT

No.	Atom	Charge	No.	Atom	Charge	No.	Atom	Charge
1	C	-0.226	14	C	-0.156	27	H	0.244
2	C	-0.244	15	C	0.552	28	H	0.247
3	C	-0.230	16	O	-0.543	29	H	0.243
4	C	-0.215	17	C	-0.154	30	H	0.253
5	C	0.160	18	C	-0.186	31	H	0.257
6	C	-0.259	19	C	-0.238	32	H	0.241
7	S	0.343	20	C	-0.212	33	H	0.239
8	C	-0.210	21	C	-0.236	34	H	0.243
9	C	0.178	22	C	-0.197	35	H	0.249
10	N	-0.450	23	C	-0.472	36	H	0.239
11	C	-0.222	24	H	0.239	37	H	0.222
12	C	-0.242	25	H	0.240	38	H	0.249
13	C	-0.192	26	H	0.246			

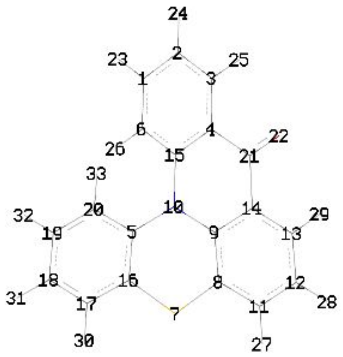

QPT

No.	Atom	Charge	No.	Atom	Charge	No.	Atom	Charge
1	C	-0.204	12	C	-0.240	23	H	0.242
2	C	-0.248	13	C	-0.177	24	H	0.242
3	C	-0.171	14	C	-0.151	25	H	0.261
4	C	-0.160	15	C	0.192	26	H	0.252
5	C	0.147	16	C	-0.209	27	H	0.249
6	C	-0.261	17	C	-0.227	28	H	0.245
7	S	0.349	18	C	-0.232	29	H	0.262
8	C	-0.217	19	C	-0.227	30	H	0.249
9	C	0.197	20	C	-0.245	31	H	0.243
10	N	-0.403	21	C	0.529	32	H	0.243
11	C	-0.213	22	O	-0.573	33	H	0.254

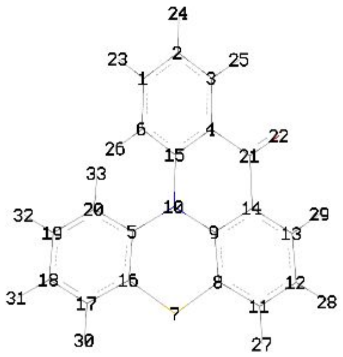

QPT$^{\bullet+}$

No.	Atom	Charge	No.	Atom	Charge	No.	Atom	Charge
1	C	-0.204	12	C	-0.240	23	H	0.242
2	C	-0.248	13	C	-0.177	24	H	0.242
3	C	-0.171	14	C	-0.151	25	H	0.261
4	C	-0.160	15	C	0.192	26	H	0.252
5	C	0.147	16	C	-0.209	27	H	0.249
6	C	-0.261	17	C	-0.227	28	H	0.245
7	S	0.349	18	C	-0.232	29	H	0.262
8	C	-0.217	19	C	-0.227	30	H	0.249
9	C	0.197	20	C	-0.245	31	H	0.243
10	N	-0.403	21	C	0.529	32	H	0.243
11	C	-0.213	22	O	-0.573	33	H	0.254

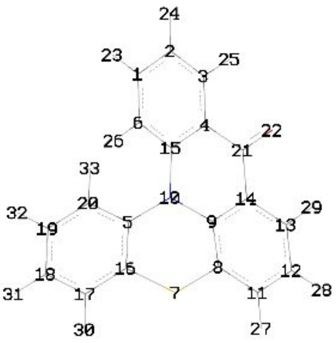

QPT•−

No.	Atom	Charge	No.	Atom	Charge	No.	Atom	Charge
1	C	-0.285	12	C	-0.249	23	H	0.213
2	C	-0.255	13	C	-0.236	24	H	0.212
3	C	-0.232	14	C	-0.128	25	H	0.242
4	C	-0.132	15	C	0.158	26	H	0.230
5	C	0.173	16	C	-0.218	27	H	0.223
6	C	-0.278	17	C	-0.239	28	H	0.215
7	S	0.280	18	C	-0.269	29	H	0.243
8	C	-0.226	19	C	-0.243	30	H	0.232
9	C	0.165	20	C	-0.254	31	H	0.222
10	N	-0.424	21	C	0.367	32	H	0.224
11	C	-0.292	22	O	-0.689	33	H	0.252

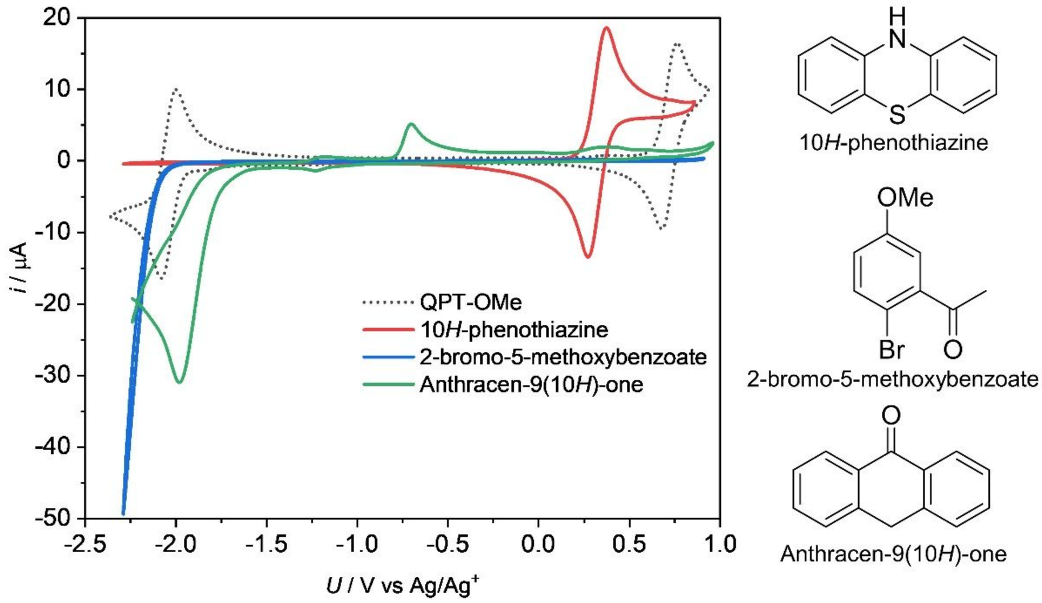

Figure S3. CV profiles of 10*H*-phenothiazine, methyl 2-bromo-5-methoxybenzoate and anthracen-9(10*H*)-one in comparison with QPT-OMe. For 10*H*-phenothiazine, the cathodic and anodic peaks centered at the potential of ca. 0.32 V vs. Ag/Ag^+, which was 0.4 V lower than those of QPT-OMe. For methyl 2-bromo-5-methoxybenzoate, the redox reaction was irreversible. There was no obvious cathodic or anodic peak in the voltage range of -2.4-1.1 V vs. Ag/Ag^+. Alternatively, we used another molecule, anthracen-9(10*H*)-one, to exclude the electron-donating effect of N in QPT-OMe. For anthracen-9(10*H*)-one, the cathodic peak appeared at the voltage of 1.98 V vs. Ag/Ag^+, which was ca. 0.15 V higher than that of the C=O in QPT-OMe.

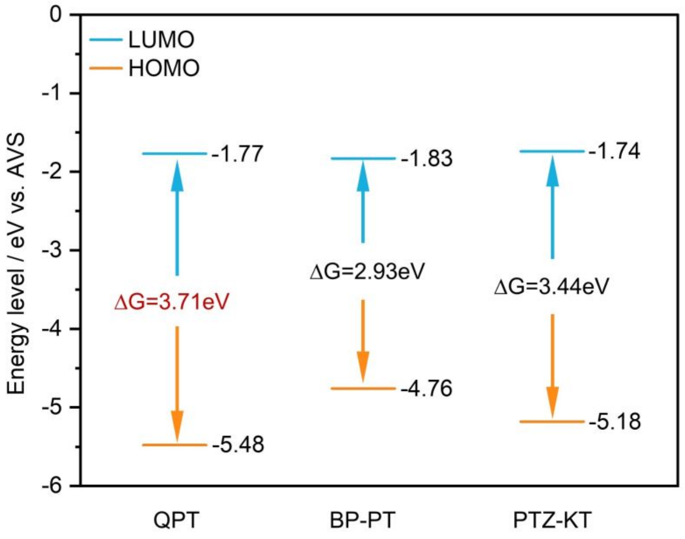

Figure S4. Frontier molecular orbital energy levels and energy gap (Δ*G*) between HOMO and LUMO of QPT, BP-PT and PTZ-KT.

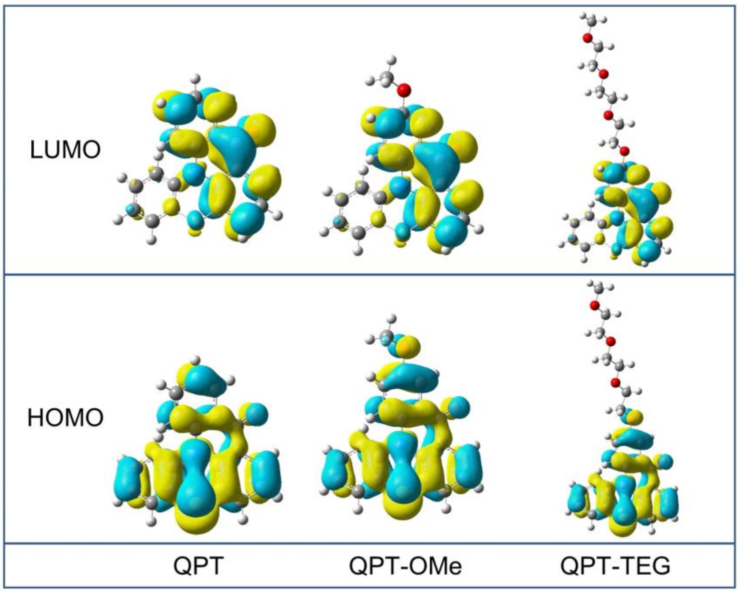

Figure S5. Molecular orbital energy level of QPT and its derivatives.

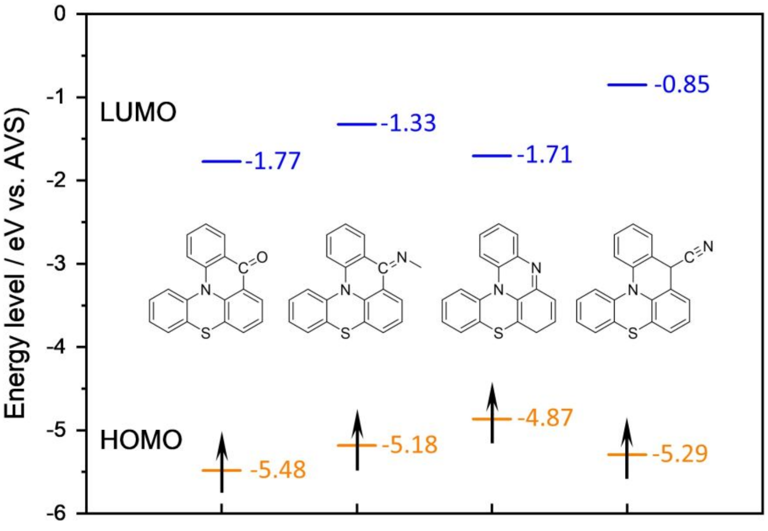

Figure S6. The HOMO and LUMO energy levels of other BRMs following the same design strategy.

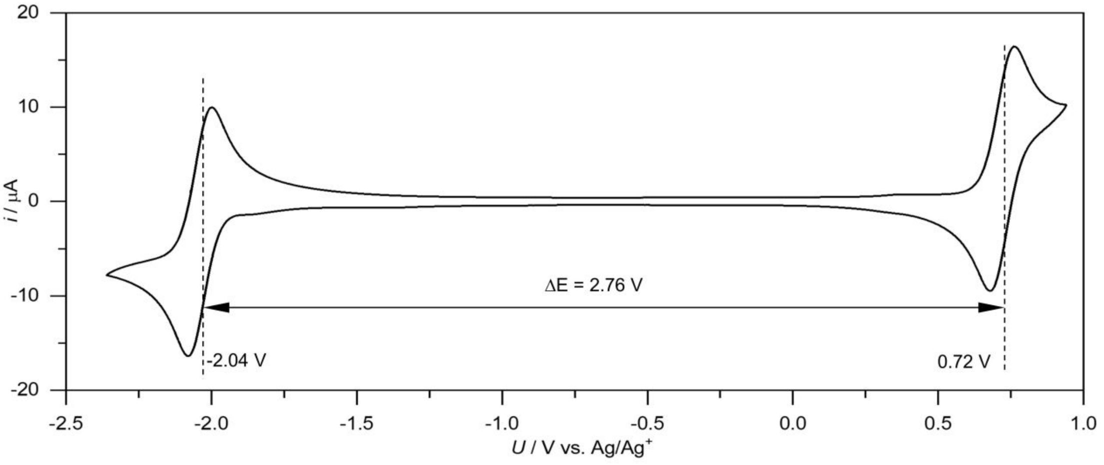

Figure S7. CV profile of 1 mM QPT-OMe and 1 mM QPT-TEG in an acetonitrile solution composed with 100 mM TBA-TFSI at sweeping rate of 50 mV s^{-1}.

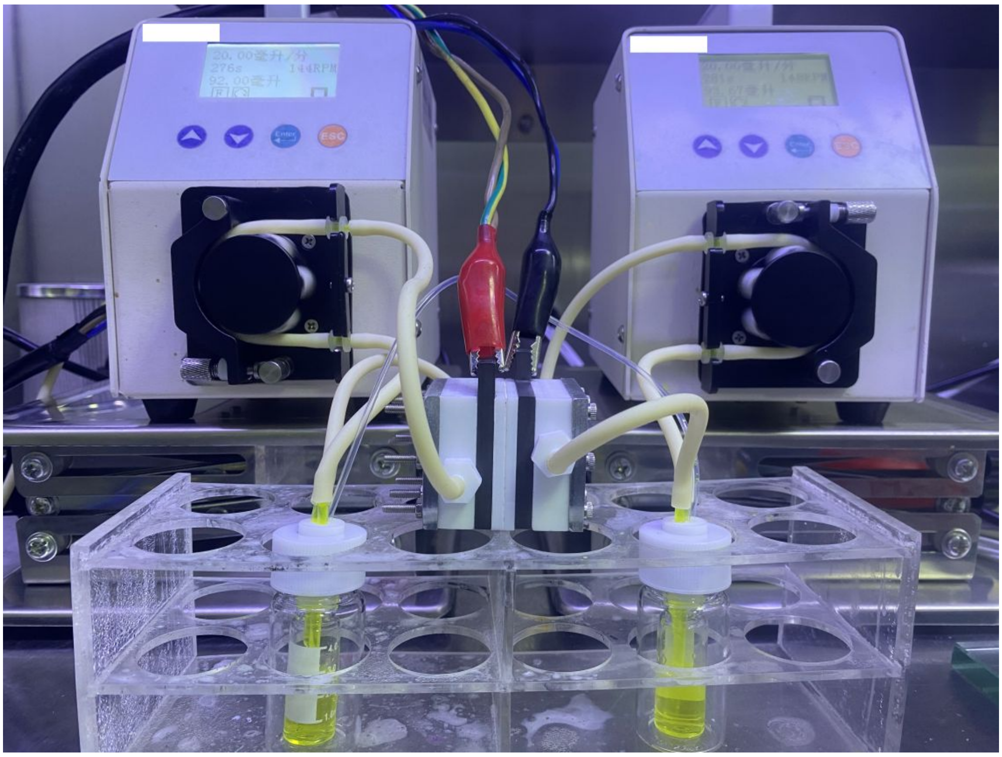

Figure S8. Digital photograph of the flow cell.

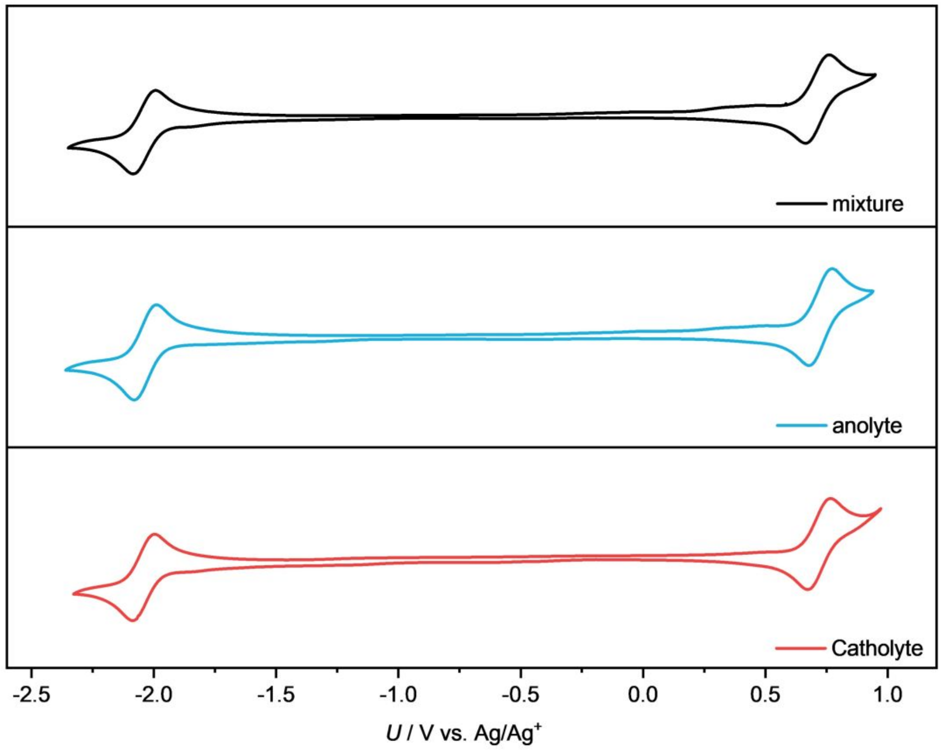

Figure S9. CV profile of the leachates of anolyte, catholyte and mixture in a non-flow cell after cycling at sweeping rate of 50 mV s^{-1}.

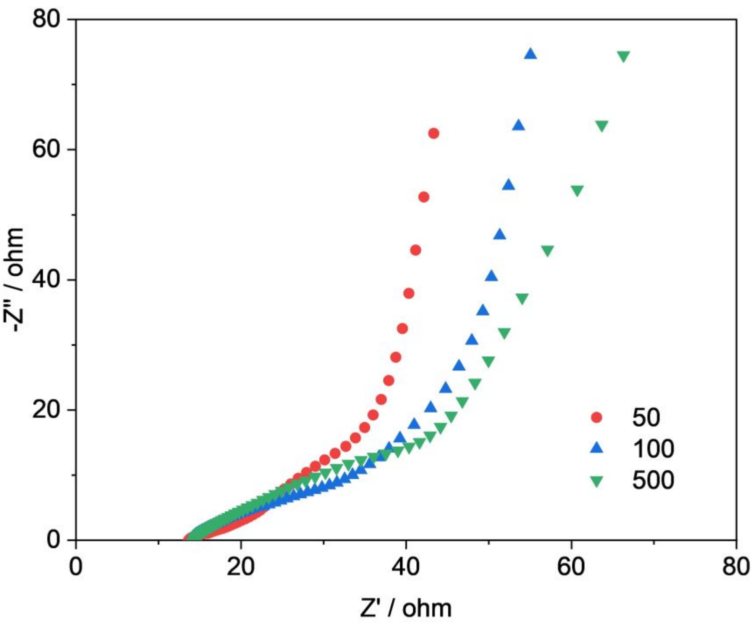

Figure S10. Electrochemical impedance spectroscopy (EIS) curves of QPT-OMe non-flow cell charge/discharge after 50, 100, and 500 cycles.

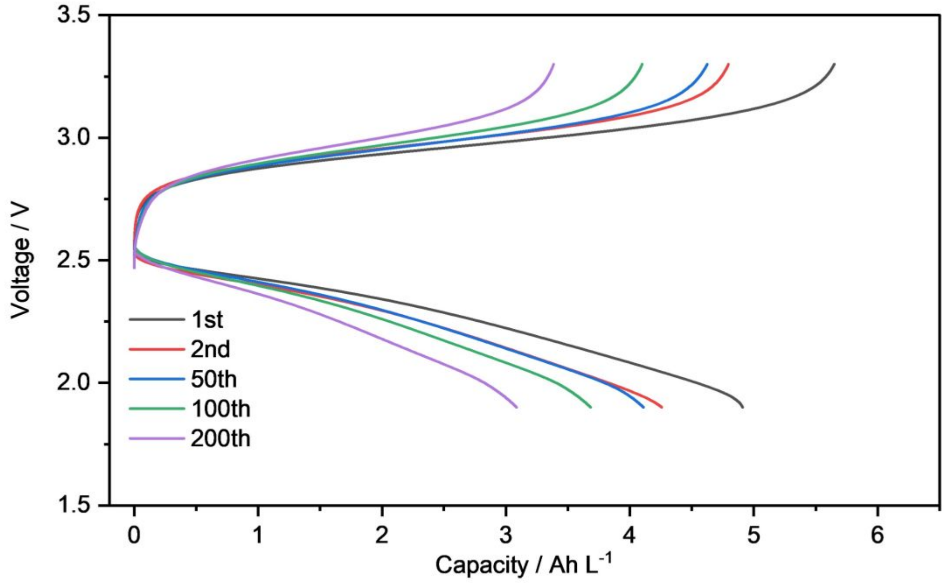

Figure S11. Representative charge-discharge profiles during long-term cycling of symmetric RFB based on QPT-TEG. The cell is evaluated at constant current density of 10 mA cm^{-2} with 0.5 M QPT-TEG and 0.5 M TBATFSI in acetonitrile as the electrolyte.

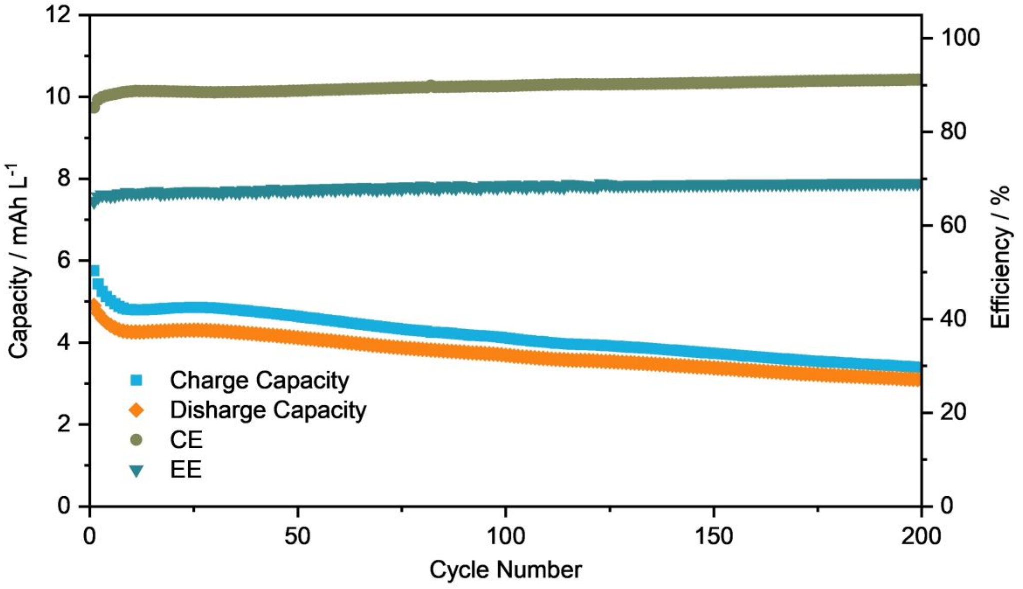

Figure S12. Corresponding capacity retention, Coulombic efficiency and energy efficiency of symmetric RFB based on QPT-TEG.

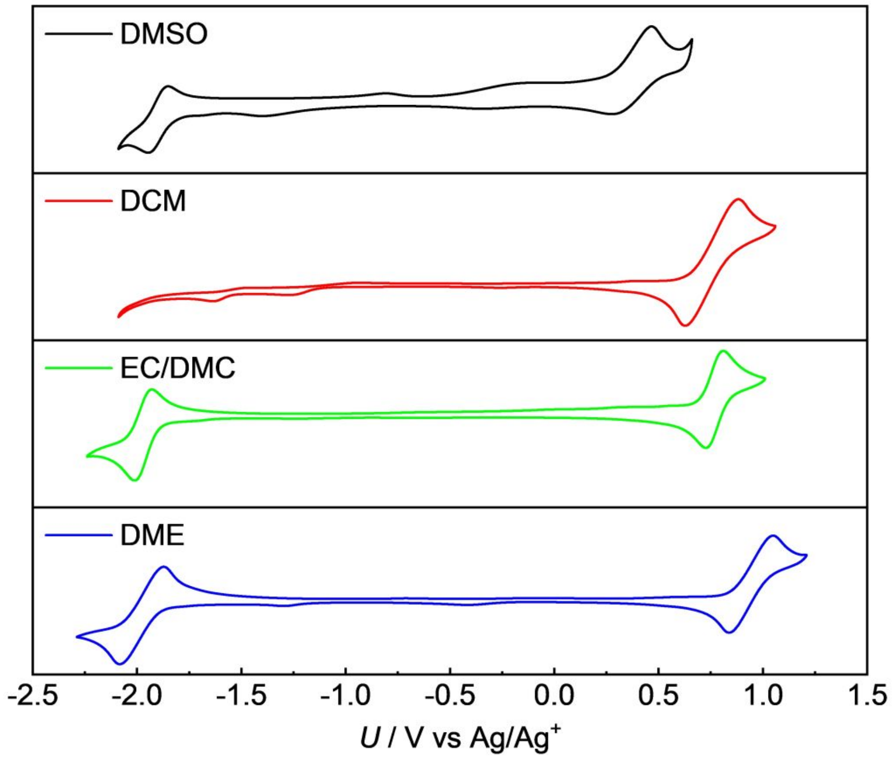

Figure S13. CV profiles of QPT-TEG in different solvents.

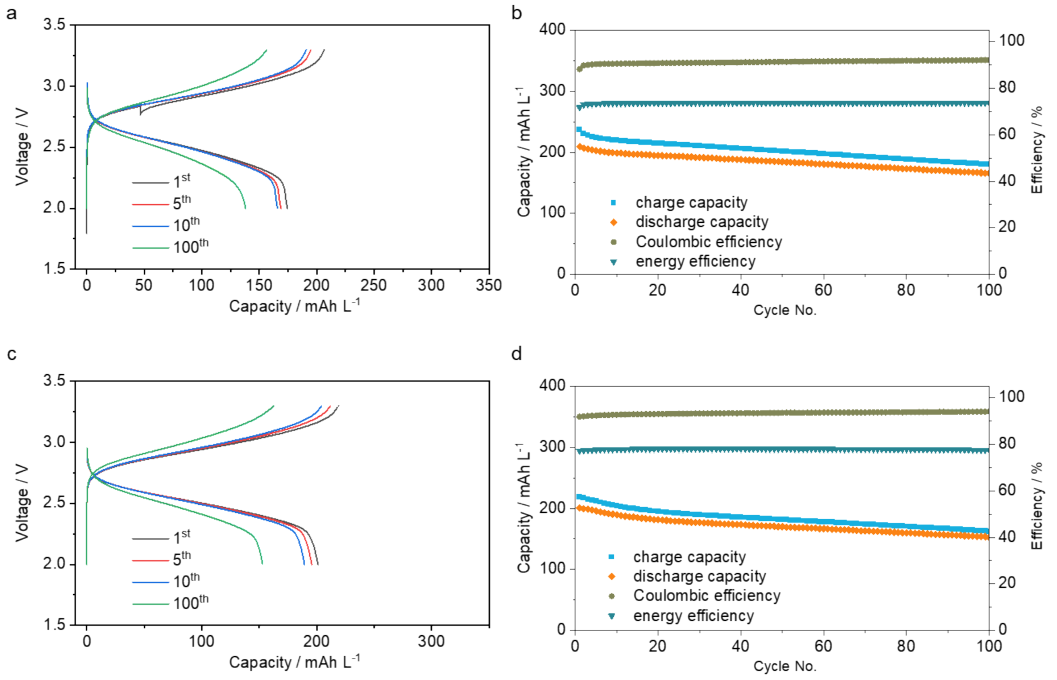

Figure S14. (a) Selected charge-discharge profiles during long-term cycling and (b) the corresponding capacity retention, CE and EE of the non-flow cell with 25 mM QPT-TEG and 0.5 M TBA-TFSI in DME as the electrolyte; (c) representative charge-discharge profiles during long-term cycling and (d) the corresponding capacity retention, CE and EE of the non-flow cell with 25 mM QPT-TEG and 0.5 M TBA-TFSI in EC/DMC as the electrolyte. The cell is evaluated at constant current density of 5 mA cm^{-2} with equal volume of 0.1 mL electrolyte in the same type of cells.

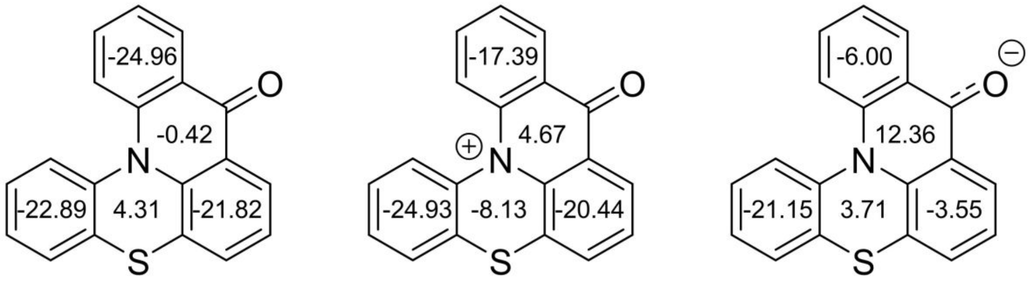

Figure S15. Nucleus-independent chemical shift (NICS) analysis of QPT, QPT$^{\bullet+}$, and QPT$^{\bullet-}$.

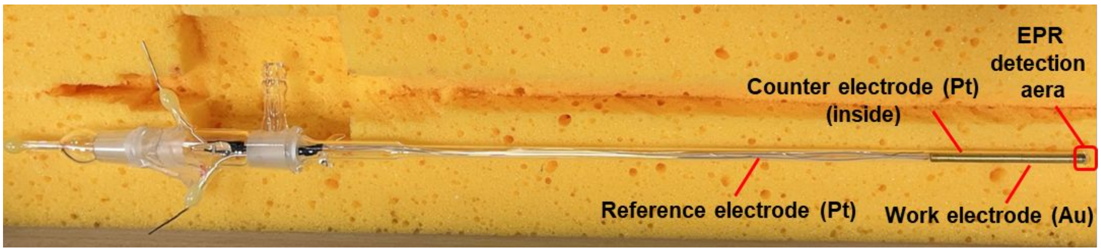

Figure S16. Customized in-situ EPR electrochemical cell for monitoring the QPT$^{\bullet+}$-OMe.

Table S2. Battery performance of reported aqueous and non-aqueous flow cells.

Anode || Cathode Materials	Solvent system	Cell Potential / V	Cycles	Ref.
viologen || TEMPO polymer	NaCl / H_2O	1.1	10000	[7]
AQDS || Br_2	HBr / H_2SO_4 / H_2O	0.9	20	[8]
TEMPO-Phenazine combi molecule symmetric	NaCl / H2O	1.2	1800	[9]
Li || TEMPO	$LiPF_6$ / (EC / PC / EMC)	3.5	100	[10]
2-MBP || DBMMB	$TEAPF_6$ / ACN	2.97	50	[11]
BzNSN || DBMMB & BzNSN-DBMMB hybrid symmetric	LiTFSI / ACN	2.36	150	[12]
Fullerene-Ferrocene hybrid molecule symmetric	NBu_4BF_4 / O-DCB	1.31 & 1.70	100	[13]
α-FcEtPI symmetric	$TBABF_4$ / 1,3-DOL	1.98	50	[14]
DAAQ symmetric	TBAP / ACN	1.76 & 2.72	6	[15]
Me-TEG-DAAQ symmetric	$TEATf_2N$ / DME	1.76 & 2.72	100	[16]
FcMeAAQ symmetric	TEATFSI / DMF	1.42	100	[17]
MEEPT symmetric	$TEABF_4$ / ACN	0.4	100	[18]
PTIO symmetric	$TBAPF_6$ / ACN	1.73	35	[19]
Nitronyl-nitroxide symmetric	$TBAPF_6$ / ACN	1.62	75	[20]

3-phenyl-1,5-di-p-tolylverdazyl symmetric	$TBAPF_6$ / ACN	0.97	50	[21]
Oxo-verdazyl radical (isoV) symmetric	TBAP / ACN	1.42	100	[22]
$Croc^{2-}$ symmetric	$TBAPF_6$ / ACN	1.82	100	[23]
H_2TPP symmetric	TBAP / DCM	2.24 & 2.83	200	[24]
BODIPY dye symmetric	$TBAPF_6$ / ACN	2.32	100	[25]
[^nPr-DMQA][BF_4] symmetric	$TBAPF_6$ / ACN	2.12	800	[26]
BuPh-DMFc	TBABF4 / ACN	1.8	500	[27]
QPT-OMe	TBATFSI / ACN	2.55	900	This work

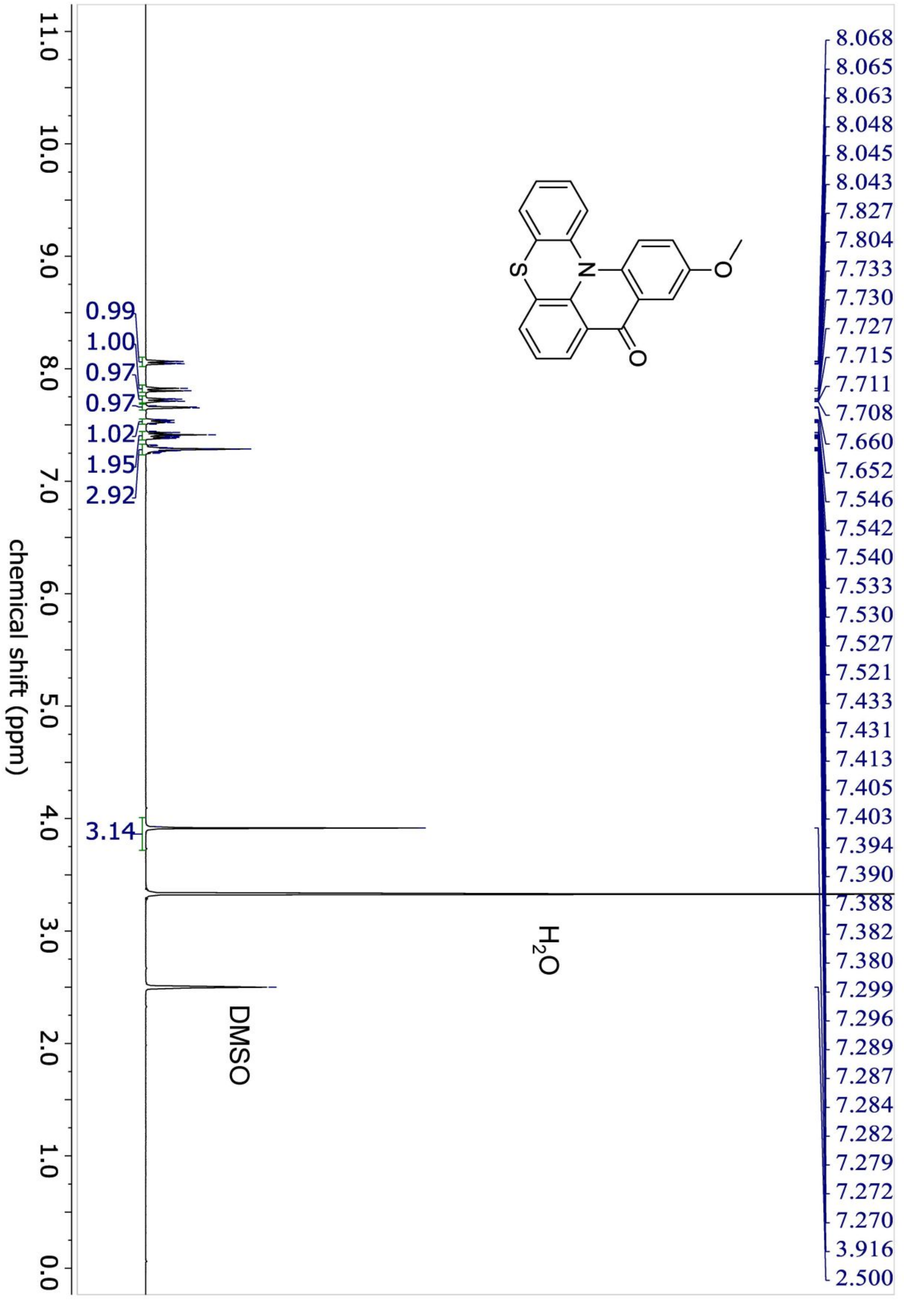

^{1}H NMR spectrum of QPT-OMe.

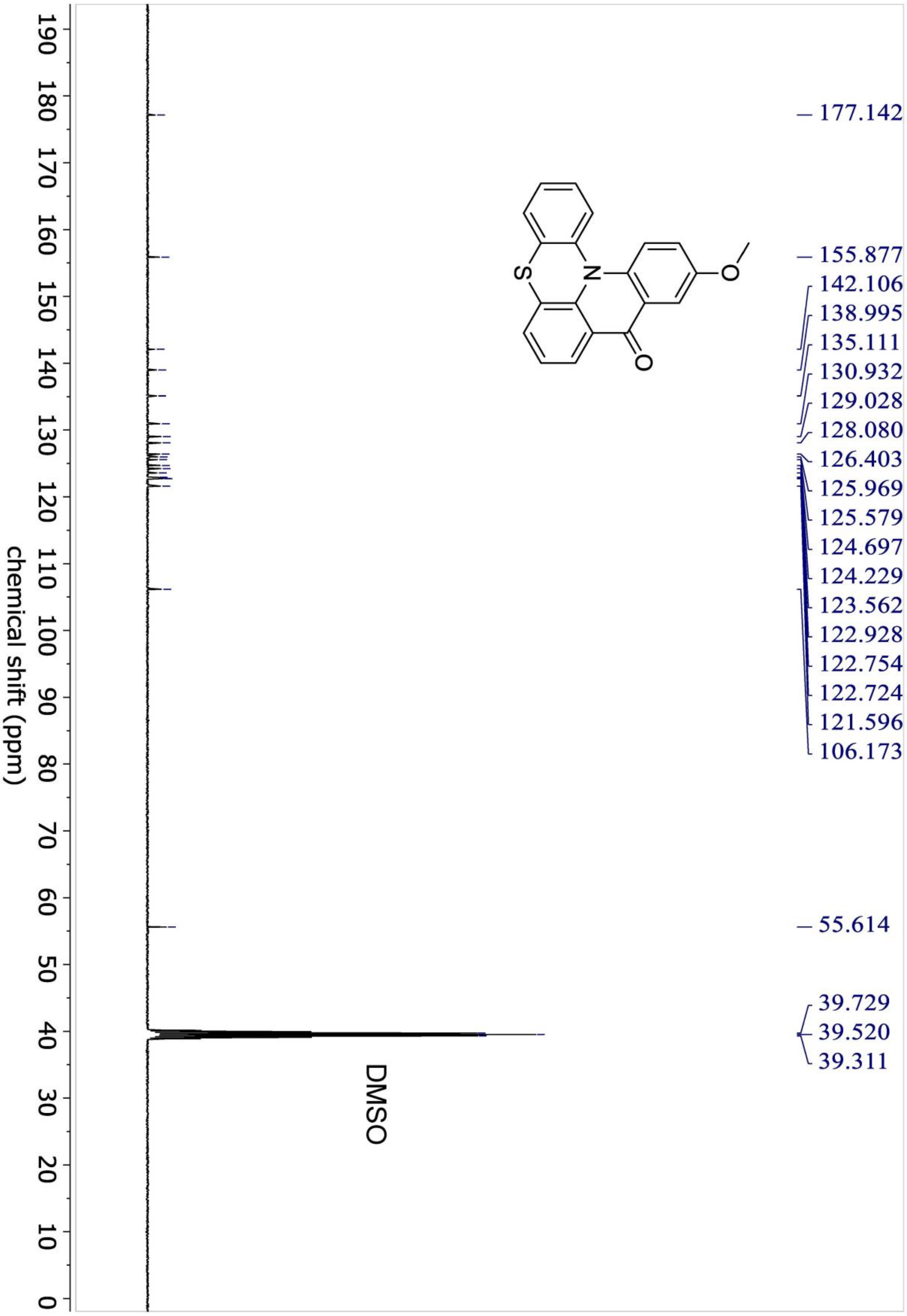

^{13}C NMR spectrum of QPT-OMe.

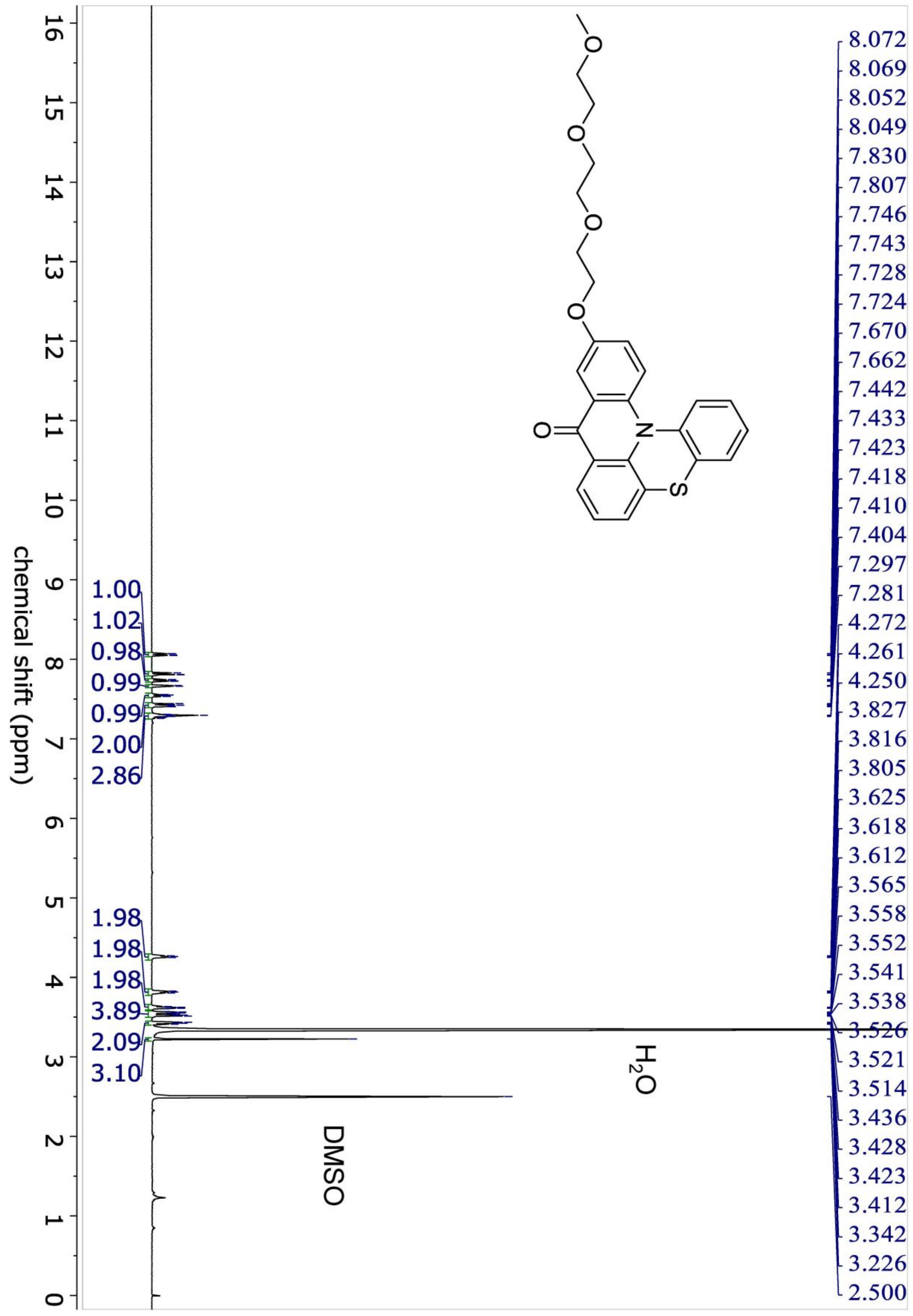

^{1}H NMR spectrum of QPT-TEG.

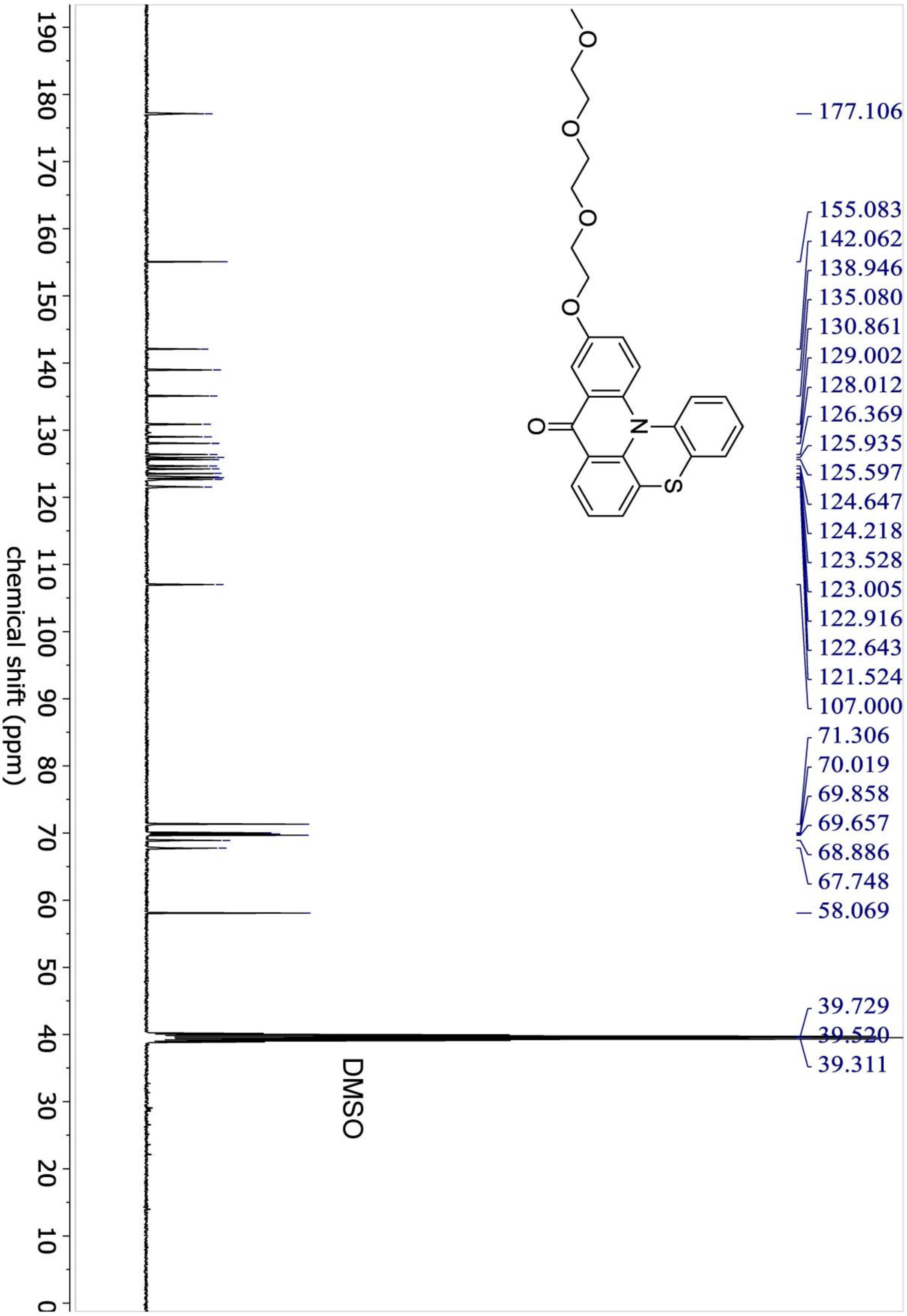

^{13}C NMR spectrum of QPT-TEG.

References

[1] Van Benschoten, J. J.; Lewis, J. Y.; Heineman, W. R.; Roston, D. A.; Kissinger, P. T., Cyclic voltammetry experiment. *J. Chem. Educ.* **1983,** *60*, 772.

[2] Ševčík, A., Oscillographic polarography with periodical triangular voltage. *Collect. Czech. Chem. Commun.* **1948,** *13*, 349-377.

[3] Randles, J. E. B., A cathode ray polarograph. Part II.-The current-voltage curves. *Trans. Faraday Soc.* **1948,** *44*, 327-338.

[4] Frisch, M.; Trucks, G.; Schlegel, H. B.; Scuseria, G. E.; Robb, M. A.; Cheeseman, J. R.; Scalmani, G.; Barone, V.; Mennucci, B.; Petersson, G., gaussian 09, Revision d. 01, Gaussian. *Inc., Wallingford CT* **2009,** *201.*

[5] Lu, T.; Chen, F., Multiwfn: A multifunctional wavefunction analyzer. *J. Comput. Chem.* **2012,** *33*, 580-592.

[6] Humphrey, W.; Dalke, A.; Schulten, K., VMD: Visual molecular dynamics. *J. Mol. Graph.* **1996,** *14* (1), 33-38.

[7] Janoschka, T.; Martin, N.; Martin, U.; Friebe, C.; Morgenstern, S.; Hiller, H.; Hager, M. D.; Schubert, U. S., An aqueous, polymer-based redox-flow battery using non-corrosive, safe, and low-cost materials. *Nature* **2015,** *527* (7576), 78-81.

[8] Huskinson, B.; Marshak, M. P.; Suh, C.; Er, S.; Gerhardt, M. R.; Galvin, C. J.; Chen, X.; Aspuru-Guzik, A.; Gordon, R. G.; Aziz, M. J., A metal-free organic-inorganic aqueous flow battery. *Nature* **2014,** *505* (7482), 195-198.

[9] Winsberg, J.; Stolze, C.; Muench, S.; Liedl, F.; Hager, M. D.; Schubert, U. S., TEMPO/phenazine combi-molecule: A redox-active material for symmetric aqueous redox-flow batteries. *ACS Energy Lett.* **2016,** *1* (5), 976-980.

[10] Wei, X.; Xu, W.; Vijayakumar, M.; Cosimbescu, L.; Liu, T.; Sprenkle, V.; Wang, W., TEMPO-based catholyte for high-energy density nonaqueous redox flow batteries. *Adv. Mater.* **2014,** *26* (45), 7649-7653.

[11] Xing, X.; Liu, Q.; Xu, W.; Liang, W.; Liu, J.; Wang, B.; Lemmon, J. P., All-liquid electroactive materials for high energy density organic flow battery. *ACS Appl. Energy Mater.* **2019,** *2* (4), 2364-2369.

[12] Duan, W.; Huang, J.; Kowalski, J. A.; Shkrob, I. A.; Vijayakumar, M.; Walter, E.; Pan, B.; Yang, Z.; Milshtein, J. D.; Li, B.; Liao, C.; Zhang, Z.; Wang, W.; Liu, J.; Moore, J. S.; Brushett, F. R.; Zhang, L.; Wei, X., "Wine-dark sea" in an organic flow battery: Storing negative charge in 2,1,3-benzothiadiazole radicals leads to improved cyclability. *ACS Energy Lett.* **2017,** *2* (5), 1156-1161.

[13] Friedl, J.; Lebedeva, M. A.; Porfyrakis, K.; Stimming, U.; Chamberlain, T. W., All-fullerene-based cells for nonaqueous redox flow batteries. *J. Am. Chem. Soc.* **2018,** *140* (1), 401-405.

[14] Hwang, S.; Kim, H.-s.; Ryu, J. H.; Oh, S. M., N-(α-ferrocenyl)ethylphthalimide as a single redox couple for non-aqueous flow batteries. *J. Power Sources* **2019,** *421*, 1-5.

[15] Potash, R. A.; McKone, J. R.; Conte, S.; Abruña, H. D., On the benefits of a symmetric redox flow battery. *J. Electrochem. Soc.* **2016,** *163* (3), A338-A344.

[16] Geysens, P.; Li, Y.; Vankelecom, I.; Fransaer, J.; Binnemans, K., Highly soluble 1,4-diaminoanthraquinone derivative for nonaqueous symmetric redox flow batteries. *ACS Sustainable Chem. Eng.* **2020,** *8* (9), 3832-3843.

[17] Zhen, Y.; Zhang, C.; Yuan, J.; Zhao, Y.; Li, Y., Ferrocene/anthraquinone based bi-redox molecule for symmetric nonaqueous redox flow battery. *J. Power Sources* **2020,** *480*, 229132.

[18] Milshtein, J. D.; Kaur, A. P.; Casselman, M. D.; Kowalski, J. A.; Modekrutti, S.; Zhang, P. L.; Harsha Attanayake, N.; Elliott, C. F.; Parkin, S. R.; Risko, C.; Brushett, F. R.; Odom, S. A., High current density, long duration cycling of soluble organic active species for non-aqueous redox flow batteries. *Energy Environ. Sci.* **2016,** *9* (11), 3531-3543.

[19] Duan, W.; Vemuri, R. S.; Milshtein, J. D.; Laramie, S.; Dmello, R. D.; Huang, J.; Zhang, L.; Hu, D.; Vijayakumar, M.; Wang, W.; Liu, J.; Darling, R. M.; Thompson, L.; Smith, K.; Moore, J. S.; Brushett, F. R.; Wei, X., A symmetric organic-based nonaqueous redox flow battery and its state of charge diagnostics by FTIR. *J. Mater. Chem. A* **2016,** *4* (15), 5448-5456.

[20] Hagemann, T.; Winsberg, J.; Häupler, B.; Janoschka, T.; Gruber, J. J.; Wild, A.; Schubert, U. S., A bipolar nitronyl nitroxide small molecule for an all-organic symmetric redox-flow battery. *NPG Asia Materials* **2017,** *9* (1), e340.

[21] Charlton, G. D.; Barbon, S. M.; Gilroy, J. B.; Dyker, C. A., A bipolar verdazyl radical for a symmetric all-organic redox flow-type battery. *J. Energy Chem.* **2019,** *34*, 52-56.

[22] Korshunov, A.; Milner, M. J.; Grünebaum, M.; Studer, A.; Winter, M.; Cekic-Laskovic, I., An oxo-verdazyl radical for a symmetrical non-aqueous redox flow battery. *J. Mater. Chem. A* **2020,** *8* (42), 22280-22291.

[23] Armstrong, C. G.; Hogue, R. W.; Toghill, K. E., Application of the dianion croconate violet for symmetric organic non-aqueous redox flow battery electrolytes. *J. Power Sources* **2019,** *440*, 227037.

[24] Ma, T.; Pan, Z.; Miao, L.; Chen, C.; Han, M.; Shang, Z.; Chen, J., Porphyrin-based symmetric redox-flow batteries towards cold-climate energy storage. *Angew. Chem. Int. Ed.* **2018,** *57* (12), 3158-3162.

[25] Kosswattaarachchi, A. M.; Friedman, A. E.; Cook, T. R., Characterization of a BODIPY dye as an active species for redox flow batteries. *ChemSusChem* **2016,** *9* (23), 3317-3323.

[26] Moutet, J.; Veleta, J. M.; Gianetti, T. L., Symmetric, robust, and high-voltage organic redox flow battery model based on a helical carbenium ion electrolyte. *ACS Appl. Energy Mater.* **2021,** *4* (1), 9-14.

[27] Zhang, C.; Qian, Y.; Ding, Y.; Zhang, L.; Guo, X.; Zhao, Y.; Yu, G., Biredox eutectic electrolytes derived from organic redox-active molecules: High-energy storage systems. *Angew. Chem. Int. Ed.* **2019,** *58* (21), 7045-7050.
